# The Therapeutic Profile of Rolipram, PDE Target and Mechanism of Action as a Neuroprotectant following Spinal Cord Injury

**DOI:** 10.1371/journal.pone.0043634

**Published:** 2012-09-19

**Authors:** Sandra Marie Schaal, Maneesh Sen Garg, Mousumi Ghosh, Lilie Lovera, Michael Lopez, Monal Patel, Jack Louro, Samik Patel, Luis Tuesta, Wai-Man Chan, Damien Daniel Pearse

**Affiliations:** 1 The Miami Project to Cure Paralysis, University of Miami Miller School of Medicine, Miami, Florida, United States of America; 2 The Neuroscience Program, University of Miami, Miami, Florida, United States of America; 3 The Department of Neurological Surgery, University of Miami Miller School of Medicine, Miami, Florida, United States of America; 4 The Interdisciplinary Stem Cell Institute, University of Miami Miller School of Medicine, Miami, Florida, United States of America; Universidad de Castilla-La Mancha, Spain

## Abstract

The extent of damage following spinal cord injury (SCI) can be reduced by various neuroprotective regimens that include maintaining levels of cyclic adenosine monophosphate (cyclic AMP), via administration of the phosphodiesterase 4 (PDE4) inhibitor Rolipram. The current study sought to determine the optimal neuroprotective dose, route and therapeutic window for Rolipram following contusive SCI in rat as well as its prominent PDE target and putative mechanism of protection. Rolipram or vehicle control (10% ethanol) was given subcutaneously (s.c.) daily for 2 wk post-injury (PI) after which the preservation of oligodendrocytes, neurons and central myelinated axons was stereologically assessed. Doses of 0.1 mg/kg to 1.0 mg/kg (given at 1 h PI) increased neuronal survival; 0.5 mg to 1.0 mg/kg protected oligodendrocytes and 1.0 mg/kg produced optimal preservation of central myelinated axons. Ethanol also demonstrated significant neuronal and oligo-protection; though the preservation provided was significantly less than Rolipram. Subsequent use of this optimal Rolipram dose, 1.0 mg/kg, via different routes (i.v., s.c. or oral, 1 h PI), demonstrated that i.v. administration produced the most significant and consistent cyto- and axo- protection, although all routes were effective. Examination of the therapeutic window for i.v. Rolipram (1.0 mg/kg), when initiated between 1 and 48 h after SCI, revealed maximal neuroprotection at 2 h post-SCI, although the protective efficacy of Rolipram could still be observed when administration was delayed for up to 48 h PI. Importantly, use of the optimal Rolipram regimen significantly improved locomotor function after SCI as measured by the BBB score. Lastly we show SCI-induced changes in PDE4A, B and D expression and phosphorylation as well as cytokine expression and immune cell infiltration. We demonstrate that Rolipram abrogates SCI-induced PDE4B1 and PDE4A5 production, PDE4A5 phosphorylation, MCP-1 expression and immune cell infiltration, while preventing post-injury reductions in IL-10. This work supports the use of Rolipram as an acute neuroprotectant following SCI and defines an optimal administration protocol and target for its therapeutic application.

## Introduction

Spinal cord injury (SCI) can occur in response to a broad range of external perturbations that contuse, compress or transect the spinal cord. The ensuing pathology often produces irreversible neuronal loss and axonal injury that culminates in permanent functional impairment [1]. Cells that survive the initial mechanical trauma are then exposed over hours to weeks to a hostile injury environment with excitotoxic molecules, pro-inflammatory cytokines, hypoxia, and oxidative stress, all of which contribute to secondary tissue damage [2], [3]. Cell death following experimental SCI has been shown to be reduced by various acute interventions [4] including anti-inflammatory and steroidal drugs [5], [6], growth factors [7], protease inhibitors [8], anti-oxidants or inhibitors of oxidative stress [5], [8] hypothermia [9] and/or cell transplantation [10], [11].

A hallmark pathophysiological response in cells affected by SCI is a dramatic reduction in levels of the ubiquitous second messenger, cyclic adenosine monophosphate (cyclic AMP) [12], a critical cellular component responsible for regulating vital intracellular functions that include cell metabolism, proliferation, survival and differentiation [12], [13]. In the nervous system, cyclic AMP serves as a potent trophic signal for neurons [14] governing their survival [15], and differentiation [16], as well as their ability to direct and elongate axons [17], [18]. Because of cyclic AMP's influence on a wide variety of cellular functions, it is predicted that there would be detrimental consequences when its levels are significantly reduced following CNS trauma to either the spinal cord [19] or brain [20].

Levels of cyclic AMP in cells can be reduced by a family of enzymes called phosphodiesterases (PDEs), the only known negative regulators of cyclic nucleotide levels [21], [22]. Following their discovery [21], PDEs have since provided an attractive target for pharmacological interventions in numerous pathological conditions in which cyclic AMP elevation has been shown to be beneficial [23]–[25]. PDE4 is the most predominantly expressed cyclic AMP-specific PDE in neural tissue [26] as well as in immune cells [27]. We have previously shown that the use of a PDE4 specific inhibitor, Rolipram, prevents injury-induced reductions in cyclic AMP after acute CNS injury [19], [20] as well as facilitates significant tissue protection, anatomical repair, and functional recovery [19]. Originally designed to treat depression [28], Rolipram has also been used in both experimental models and as a clinical therapy for Asthma [29], Arthritis [30], Huntington's Disease [31], Multiple Sclerosis [32], Alzheimer's Disease [33], Human immunodeficiency virus (HIV) infection [34], and traumatic brain injury (TBI) [20]. The widespread application of this PDE inhibitor for a variety of neuropathological conditions is not only indicative of the important role of PDE4 within the nervous system, but also to the pivotal function of the cyclic AMP:PDE axis in regulating immune cell activation and inflammatory processes, which are key components of neurodegenerative disorders [27], [35].

In the current study, we have built upon earlier work [19] to determine the optimal dosing, route and therapeutic window of Rolipram as an acute neuro- and axo-protectant for SCI. In addition, we have identified the likely PDE4 target(s) of Rolipram after SCI, the reductions of which could be responsible for these protective effects. Finally, we have measured cytokine and immune cell changes that accompany and may form the basis of Rolipram-mediated neuroprotection. These studies pave the way for moving Rolipram towards clinical application and provide mechanistic data important for the future development of PDE4 therapeutics for CNS injury.

## Methods

### Ethics Statement

Animals were housed according to NIH guidelines and The Guide for the Care and Use of Animals. All animal procedures were approved by the University of Miami Miller School of Medicine Institutional Animal Care and Use Committee (IACUC).

### Animals

#### Pre-operative preparation

Adult female Fischer rats (Harlan Co. n = 337; 180–200 g) were housed according to NIH guidelines and The Guide for the Care and Use of Animals. All animal procedures were approved by the University of Miami Miller School of Medicine Institutional Animal Care and Use Committee (IACUC). Prior to surgical procedures, animals were weighed and anesthetized by intraperitoneal (i.p.) injection (45 mg/kg ketamine, 5 mg/kg xylazine). An adequate level of anesthesia was determined by monitoring the corneal and hindlimb withdrawal reflexes. The back and neck regions were then shaved and aseptically prepared with chlorhexidine (Phoenix Pharmaceutical Inc., St. Joseph, MO). Lacrilube ophthalmic ointment (Allergan Pharmaceuticals, Irvine, CA) was applied to the eyes to prevent drying. Throughout the surgery, the rats were kept on a homeothermic blanket system (Harvard Apparatus Ltd., Kent, UK) to maintain body temperature at 37+/−0.5°C as assessed by a rectal probe.

#### Jugular vein catheterization

Prior to laminectomy and injury induction, in those animals receiving intravenous (i.v.) Rolipram or vehicle, a sterile heparinized catheter consisting of PE50 tubing (Becton Dickinson and Company, Sparks, MD) was placed into the animal's right external jugular vein. The surgical procedure was performed as described previously [36]. Briefly, an incision was made between the mandible and clavicle, on the right side of the sternum, and superficial tissue was bluntly dissected to reveal the external jugular vein. An incision was made in the vein and the catheter was directed caudally for 1 cm into the vein. Once placed, the i.v. line was flushed with 0.9% physiological saline and functionally checked by blood withdrawal. The animal was then placed in a prone position and a superficial incision was made on the dorsal surface of the neck. Hemostats were used to lace the tubing along the posterior of the animal, through the superficial soft tissue of the back and neck. The catheter was then pulled out of the back, shortened and then secured in place with sutures and one wound clip. The incision on the ventral surface was closed by suturing. For repetitive agent administration, the catheter tubing was aseptically capped with a sterile stainless steel wire plug between administrations.

#### Moderate thoracic contusion injury

Rats were subjected to a moderate contusion injury using the MASCIS impactor [37]. A laminectomy at thoracic vertebra T8 exposed the dorsal surface of the spinal cord underneath without disrupting the dura mater. Stabilization clamps were placed around the vertebrae at T6 and T12 to support the column during impact. The exposed spinal cord was moderately injured by dropping a 10.0 g rod from a height of 12.5 mm. The contusion impact height, velocity and compression were monitored. Animals (n = 12) were excluded immediately when height or velocity errors exceeded 7% or if the compression distance was not within the range of 1.25 to 1.75 mm. After injury, the muscles were sutured in layers and the skin was closed with metal wound clips. The rats were allowed to recover in a warmed cage with water and food easily accessible. Gentamicin (5 mg/kg, intramuscular; Abbott Laboratories, North Chicago, IL) was administered immediately post-surgery and then daily for seven days. The analgesic, Buprenex (0.01 mg/kg, s.c.; Reckitt Benckiser, Richmond, VA) was delivered post-surgery and daily for 2 days.

#### Rolipram administration

The phosphodiesterase inhibitor, Rolipram (Sigma, St. Louis, MO), was first dissolved in 100% ethanol and then gently mixed with 0.9% physiological saline to a final 10% v/v solution. Ten percent ethanol (in physiological saline) therefore served as the vehicle for the control group. According to the animal's specific study group (Table 1), they were administered Rolipram or vehicle once daily for a period of two weeks. In initial dose-finding studies, animals received s.c. administration of 250 µl Rolipram (0.1 to 5.0 mg/kg) or vehicle beginning at 1 h post-SCI. In the subsequent optimal route study, 250 µl of 1.0 mg/kg Rolipram or vehicle was administered orally, s.c. or i.v at 1 h post-SCI. In the final therapeutic window study, animals received 250 µl of 1.0 mg/kg Rolipram or vehicle i.v. beginning at 2 to 48 h post-SCI. Studies with biochemical and behavioral endpoints used the optimal regimen for Rolipram (1.0 mg/kg i.v. at 1 h post-SCI), except where specifically indicated (Table 1). For oral administration, the rats received Rolipram or vehicle via a Kendall (Mansfield, MA) monoject tuberculin syringe into the oral cavity. Subcutaneous delivery involved an injection below the skin on the dorsal region of the animal at the right flank, an area distant to the injury site. For the treatment group receiving i.v. administration, Rolipram or vehicle (250 µl volume) was slowly delivered through the i.v. line over 2 minutes, followed by a flush of 250 µl of physiological saline to ensure complete delivery. Treatment groups for each experimental aim and the number of animals used for the various outcome measures are delineated in Table 1. During the studies detailed, there were 7 pre-endpoint deaths. Four animals died during anesthesia or surgical procedures and three animals were found dead during the post-injury survival period; Study 1 (1×5.0 mg/kg s.c. Rolipram) and Study 9 (1× SCI control and 1× vehicle control).

**Table 1:** Experimental outline of treatment groups, time points and sample numbers.

STUDY ID	STUDY OUTCOME/MEASURE	TREATMENT GROUPS (FINAL ‘N’)						
**1**	0ptimal Dose[*]	Injury-only (n = 5)	Vehicle (n = 7)	0.1 mg/kg (n = 5)	0.5 mg/kg (n = 5)	1.0 mg/kg (n = 5)	5.0 mg/kg (n = 4)	
**2**	Optimal Route[*]	Injury-only (n = 5)	Vehicle i.v (n = 7)Vehicle s.c. (n = 7)	1.0 mg/kg Oral (n = 6)	1.0 mg/kg Subcut. (n = 5)	1.0 mg/kg Intravenous (n = 5)		
**3**	Optimal therapeutic window[* **,** $]	Injury-only (n = 5)	Vehicle (n = 9)	1 h PI (n = 6)	2 h PI (n = 5)	4 h PI (n = 6)	24 h PI (n = 6)	48 h PI (n = 6)
**4**	Cytokine protein array[$ **^,^** +]	Uninjured (n = 3)	Injury- only (n = l2)	Vehicle (n = 12)	1 h PI (n = 4)	4 h PI (n = 4)	25 h/1 h PI (n = 4)	25 h/24 h PI (n = 4)
**5**	Cytokine ELlSAs[$ **^,^** +]	Uninjured (n = 3)	Injury- only (n = 6)	Vehicle (n = 6)	1 h PI (n = 3)	25 h/24 h PI (n = 3)		
**6**	PDE4 Immunoblots[$ **^,^** +]	Uninjured (n = 4)	Injury- only (n = l2)	Vehicle (n = 12)	2 h/1 h PI (n = 5)	4 h/1 h PI (n = 5)	25 h/1 h PI (n = 5)	25 h/24 h PI (n = 5)
**7**	PDE4 immunohistochemistry [24 h post-SCI survival$]	Uninjured (n = 4)	Injury- only (n = 5)	Vehicle (n = 5)	1 h PI (n = 5)			
**8**	Immune cell infiltration[$ **^,^** #]	Injury only (n = 7)	Vehicle (n = 7)	1 h PI (n = 7)	2 h PI (n = 8)	4 h PI (n = 7)	24 h PI (n = 10)	48 h PI (n = 8)
**9**	Locomotor Function[$ **^,^** &]	Injury only (n = 8)	Vehicle (n = 8)	1 h PI (n = 8)				
	**TOTAL**	**318+19 (attrition/exclusion) = 337 rats**						

The animals used in this study were divided into 9 separate experiments based on the outcomes of the work. Listed in the table are the goals of each experiment as well as the treatment group allocation and number of animals per group. Gray shading is used to identify Rolipram treatment groups. Timepoints for specific groups indicate the sacrifice endpoint; when separated by a “/” the second time indicates the time when Rolipram was delivered. Symbols are as follows:

(*) animals survived for 2 weeks post-SCI;

(#) animals survived for 3 days post-SCI;

(&) animals survived for 6 weeks post-SCI;

($) animals given 1.0 mg/kg Rolipram i.v.;

(+) injury only and vehicle controls sub-divided into 3 groups (1, 4, or 25 h post-SCI; n = 4 each).

### Biochemistry

#### Tissue preparation

At 2 to 25 h after injury, animals were deeply anesthetized (70 mg/kg ketamine, 10 mg/kg xylazine), decapitated and spinal cord segments (5 mm), encompassing the injury epicenter or immediately rostral or caudal, were separately dissected. Dissected cord samples were immediately placed into cryovials, snap frozen in liquid nitrogen and then stored at −80°C until the running of one of the following assays.

#### Western blot analysis

Tissue samples were rapidly thawed at 37°C and homogenized with a Dounce homogenizer (30 strokes, 4°C) in 1 ml of ice-cold cell lysis buffer [20 mM Tris-HCl (pH 7.5), 150 mM NaCl, 1 mM EDTA, 1 mM EGTA, 1% Triton X-100, 2.5 mM sodium pyrophosphate, 1 mM β-glycerophosphate, 1 mM Na_3_VO_4_, 50 mM NaF, 1 µg/ml leupeptin, 1 mM PMSF, and 1× Roche's complete protease inhibitor cocktail (Roche Diagnostics GmbH, Mannheim, Germany). The samples were centrifuged (1000×g, 10 min, 4°C) and the supernatants assayed for total protein. Protein concentrations were determined by the BCA protein assay, according to the manufacturer's instructions (Bio-Rad Laboratories, Hercules, CA). Prior to immunoblotting, protein extracts were normalized to a final concentration of 1 mg/ml and boiled at 100°C for 20 min in Laemmli buffer (100 mM Tris, pH 6.8, 250 mM β-mercaptoethanol, 4% sodium dodecyl sulfate, 0.01% bromophenol blue and 20% glycerol). To assess changes in PDE4 gene product expression and phosphorylation by SDS-polyacrylamide gel electrophoresis, 20 µg of protein from each sample was loaded per lane and run on a 10% gel. Following overnight electro-transfer (room temperature, 25 V) onto a Nitrocellulose blotting membrane (PALL Life Sciences Corporation, BioTraceNT, FL), membranes were blocked in 3% BSA (fraction V; Sigma) in TBS-T (50 mM Tris-HCl, 150 mM NaCl, 0.15% Tween-20, pH 8.0) and then probed with different primary antibodies diluted 1/1000 in the blocking buffer. The primary antibodies used were rabbit polyclonals against PDE4A, PDE4B and PDE4D (1∶1000, FabGennix Inc. International, Frisco, Texas) or phosphorylated PDE4A (Serine; 1∶500, Abcam Inc., Cambridge, MA). Membranes were incubated for 2 h with the primary antibodies at room temperature followed by 1 h with a secondary anti-rabbit IgG conjugated with horseradish peroxidase (1∶1000; Jackson ImmunoResearch Laboratories, Inc. West Grove, PA). Visualization of antibody-bound protein was accomplished using the SuperSignal West Pico Chemiluminescent detection kit (Thermo Fisher Scientific Inc, Rockford, IL). The relative amount of the immunoreactive protein in each band was determined by scanning densitometric analysis of the X-ray films (Fluor-S Multi Imager, Bio-Rad). Density readings were normalized to β-actin immunoreactivity (1∶10,000, Sigma-Aldrich) performed on the same blot.

#### Cytokine protein array

A rat cytokine protein array system from RayBiotech, Inc. (Norcross, GA) was used according to manufacturer's instructions to quantify the levels of 19 different cytokines and chemokines within the injured spinal cord at 2 or 25 h after injury and either vehicle or Rolipram treatment (optimal dose; 1.0 mg/kg i.v., as described above, given at either 1 or 24 h post-SCI; n = 4/group/time point; Table 1). Uninjured animals were used as a baseline control. At the time of assay, tissue was thawed and homogenized as described above for immunoblotting. Homogenates were centrifuged at ×4,400 g for 10 min at 4°C. The supernatant containing the cytoplasmic fraction was re-suspended in 1∶10 buffer containing: 50 mM Hepes (pH 7.9), 0.75 mM MgCl_2_, 0.4 M KCl, 0.5 mM DTT, 0.5 mM EDTA, 2 mM Na_3_VO_4_, 5 mM NaF, 12.5% glycerol, and 100 µg/ml AEBSF, and then centrifuged at ×17,500 g for 10 min at 4°C. Protein level in each sample was assayed using the DC Protein Assay from Bio-Rad (Hercules, CA). Prior to addition of sample, protein array membranes were arranged in supplied eight well trays and blocked for 30 min with blocking buffer at room temperature. Homogenates (containing 3 mg of protein) were added to the membranes and incubated for 2 h at room temperature. After washing, biotin-conjugated antibodies (diluted to 0.4% in buffer) were added to each membrane and incubated at 4°C overnight. Following washing, HRP-conjugated streptavidin (diluted to 0.1% buffer) was added to each membrane and incubated at room temperature for 60 min. After washing, membranes were submerged into a mixed buffer solution and incubated at room temperature for 1 min. Membranes were excised from the trays, excess moisture removed and the membrane placed in a clear plastic cover page in preparation for exposure. The signal for each cytokine and chemokine (in duplicate) was detected by chemiluminescent film and the intensity quantified by densitometry using Quantity-One software for the Fluor-S MultiImager equipment from Bio-Rad. The average signal intensity for each cytokine, treatment and time point, normalized via the provided positive control, was compared to its respective injured, vehicle and injury only controls.

#### ELISA

Spinal cord samples from the injury epicenter or rostral or caudal cord were first weighed and a 10 times volume of cell lysis buffer (Cell Signaling Technology, Danvers, MA) containing a Complete™ protease inhibitor tablet (Roche, Penzberg, Germany) was added to the samples on ice. Samples were homogenized at 4°C for 45 seconds using a Polytron homogenizer and then centrifuged at 5,700 g for 10 min at 4°C. The supernatant, constituting the cytosolic fraction, was separated and the pellet containing cellular debris discarded. All supernatants were stored at −80°C until use. To determine protein concentration within each sample, the BCA protein assay [Bio-Rad Laboratories (Hercules, CA)] was performed using a microplate reader (Molecular Devices, SpectraMax, M5) on a 96-well plate with 5 µl of supernatant. Samples were then diluted in Diluent B of the MCP-1 or IL-10 ELISA kits and run as duplicates according to the manufacturer's instructions (RayBiotech, Inc. Norcross, GA). Final concentrations of MCP-1 and IL-10 were normalized to the protein concentration of the sample.

### Histology

#### Transcardial perfusion and tissue preparation

**Figure 1:**
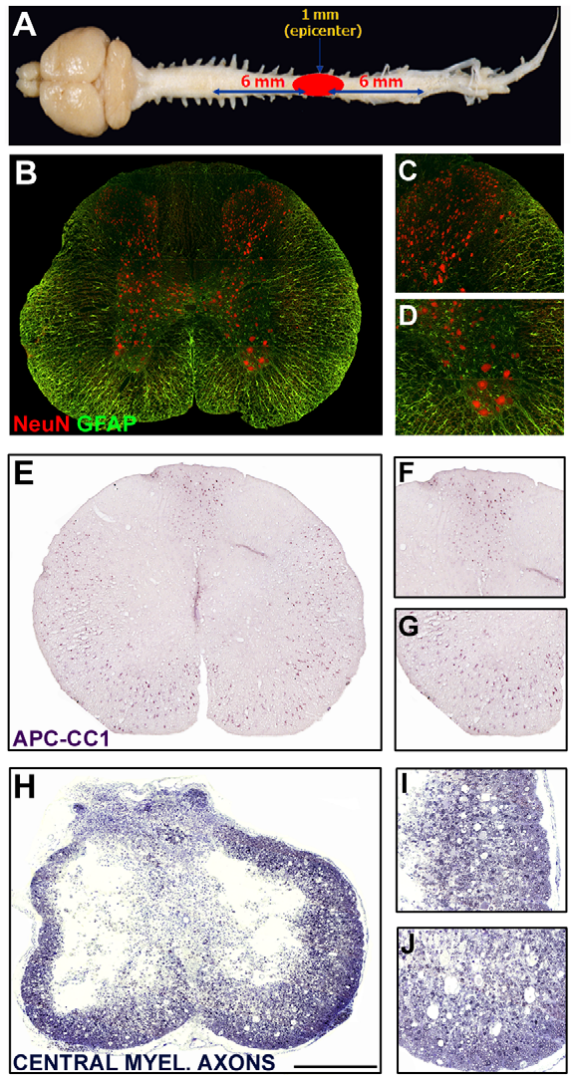
Spatial schematics for stereological quantification of preserved neurons, oligodendrocytes and central myelinated axons. **A.** Dissected CNS tissue was processed such that a 1 mm-long piece from the center of the injury was removed for plastic embedding and the preparation of transverse semithin sections. These sections were stained with toluidine blue to permit identification and quantification of central myelinated axons. Segments of spinal cord 6 mm rostral and caudal to the excised center block were paraffin embedded, sectioned transversely and used for immunohistochemical identification and stereological quantification of NeuN^+^ neurons and APC-CC1^+^ oligodendrocytes. Please note that the indicated measurements for the excised region indicated upon the spinal cord are not drawn to scale. **B–D.** NeuN^+^ neurons within a transverse spinal cord section at 6 mm from the injury site (**B**) with higher magnification images of the dorsal (**C**) and ventral horns (**D**). Preserved neurons were quantified in all regions. **E–G.** APC-CC1^+^ oligodendrocytes within a transverse spinal cord section at 6 mm from the injury site (**E**) with higher magnification images of the dorsal (**F**) and ventral white matter tracts (**G**). Preserved oligodendrocytes were quantified throughout all white matter regions. **H–J.** Toluidine blue stained transverse plastic section from the injury epicenter showing central cavitation and a surrounding peripheral rim of spared white matter. Examination of the lateral (**I**) and ventral (**J**) white matter regions shows evidence of preserved central myelinated axons as identified by the ring structures that are devoid of a closely associated cell nucleus. Using a Zeiss microscope equipped with Stereo Investigator software, contours were created and immunoreactive cells or stained myelin rings within these transverse sections were quantified. Sections at defined intervals across a specific length of spinal cord were analyzed and sampled data used to estimate total numbers within the structures' volume.

From 24 h to six weeks post-SCI, rats were deeply anesthetized (70 mg/kg ketamine, 10 mg/kg xylazine) and transcardially perfused first with 500 ml of ice-cold physiological saline and then with 4% paraformaldehyde (PFA, 0.1 M, pH 7.4; [38]). The brain and spinal cord were then dissected and post-fixed in paraformaldehyde for five days at 4°C (paraffin) or overnight followed by 48 h in 30% w/v sucrose (tissue-tek). For spinal cord pieces that were to be used for paraffin embedding, a 1 mm block was first removed from the injury epicenter and transferred to 2% glutaraldehyde for use in the preparation of semi-thin sections (central myelinated axon counts; [39]). Then, 6 mm blocks rostral and caudal to the injury epicenter or a 2 cm length of spinal cord encompassing the injury epicenter was collected and embedded in paraffin or tissue-tek, respectively, to prevent further damage during histological procedures (See Figure 1A). The paraffin embedded blocks were transversely sectioned at 10 µm on a microtome for use in the stereological counting of neurons (Figure 1B–D) and oligodendrocytes (Figure 1E–G) by computer-assisted microscopy while the tissue-tek embedded blocks were transversely sectioned at 20 µm on a cryostat and used to determine the cellular localization of PDE4 gene products. Every fourth section was collected during sectioning of the paraffin blocks and mounted in 5 series (each section separated by a 200 µm interval) onto Snowcoat X-tra slides (Surgipath, Winnipeg, Manitoba) and stored until required for immunohistochemistry. While for tissue-tek blocks, every section was collected during sectioning and mounted in 10 series (each section separated by a 200 µm interval) onto Snowcoat X-tra slides.

#### Semi-thin section preparation

The 1-mm portion of spinal cord encompassing the injury epicenter was saved for plastic embedding to generate semi-thin sections. After post-fixation, the blocks were transferred to 2% glutaraldehyde and prepared as previously described [40]. The 1-mm block was then cut into 1-µm transverse semi-thin sections, stained with 1% toluidine blue- 1% methylene blue- 1% sodium borate so as to permit the counting of spared, central myelinated axons (Figure 1H–J).

#### Immunohistochemistry

To identify neuronal cells, oligodendrocytes, activated immune cells or PDE4 gene products in collected transverse sections, tissues were immunohistochemically stained in separate series following a previously described protocol [41]. For identification and stereological quantification of neuronal perikarya or activated immune cells, sections were first de-paraffinized, dehydrated and incubated for 20 min in citrate buffer at 96°C for antigen retrieval. Once cooled to room temperature, sections were rinsed in TBS, blocked with 5% HINGS in TBS for 1 h at room temperature and then incubated overnight at 4°C with a neuron-specific monoclonal antibody, NeuN (1∶200; Chemicon, Temecula, CA) or anti-rat ED1 monoclonal antibody (1∶200; AbD Serotec, Raleigh, NC) for macrophages [42] as well as a polyclonal antibody for glial fibrillary acidic protein (GFAP) (1∶1000; DAKO Cytomation, Carpinteria, CA) to label astrocytes for spinal cord architecture. For visualization, fluorescent secondary antibodies were used, Alexa 594-conjugated goat anti-mouse (Invitrogen, Carlsbad, California) and Alexa 488-conjugated goat anti-rabbit (Invitrogen). In series of sections from another cohort of animals that were cryosectioned, NeuN, the anti-CD11b (OX-42; 1∶200, Becton, Dickinson and Company, Franklin Lakes, NJ) monoclonal antibody for macrophages or the APC-CC1 monoclonal antibody (1∶50, EMD Chemicals, San Diego, CA), specific for oligodendrocytes [43], was combined not with GFAP but with rabbit polyclonal antibodies against PDE4A, PDE4B or PDE4D (1∶100, FabGennix Inc. International, Frisco, Texas) or phosphorylated PDE4A (Serine; 1∶100, Abcam Inc., Cambridge, MA) using the same protocol. The PDE4 antibodies (FabGennix Inc.) used in the current study have been shown to specifically recognize PDE4 spliced variants for each of the gene products examined [44]–[46]. Non-specific binding was controlled for using primary and secondary antibody only controls. After staining, all sections were incubated with the nuclear counterstain Hoechst (1∶500, Sigma). The sections were cover-slipped with Vectashield mounting medium (Vector Laboratories Inc., Burlingame, CA) for microscopic evaluation. To quantify numbers of preserved oligodendrocytes, APC/CC1 (1∶100) was used on a series of sections from the paraffin-embedded tissue used for NeuN∶GFAP and ED1∶GFAP staining. Following overnight incubation with APC/CC1 (1∶50) the slides were subjected to a biotin conjugated goat-anti mouse secondary antibody (Sigma). After streptavidin incubation for 1 h, immunoreactivity was visualized with Vector-VIP (Vector Laboratories Inc.), a purple chromogen, as previously described [47] and slides were coverslipped using VectaMount (Vector Laboratories Inc.).

### Imaging and Stereology

Images were taken using the 20×, 40× or 60× objective of an Olympus BX51 microscope with meander scan software (MicroBright Field Inc.). The tonal range and sharpness (smart sharpen, 1.9 pixels) of the Tiff files were then normalized in range using Adobe CS2 (Adobe Systems Inc., San Jose, CA).

#### Quantification of Neurons, Oligodendrocytes and Macrophages

The number of NeuN^+^ neurons within the entirety of the gray matter or CC1^+^ oligodendrocytes within spared peripheral white matter regions was counted using the optical fractionator tool of Stereo Investigator (MicroBrightfield Inc.) under 600× magnification (Figure 1). Transverse sections at 400 µm intervals through 4 mm or 3 mm, respectively, rostrally and caudally from the injury epicenter were sampled for stereological analysis. A grid size of 100 µm×100 µm and a sampling frame of 40 µm×40 µm were applied to each transverse section. Immunoreactive NeuN^+^ neurons were identified and marked using an optical probe; probe counts were totaled [39] across the entire length of the blocks to generate the NeuN^+^ neuronal cell counts within each spinal cord sample. A total of 8–10 rostral and caudal sections were sampled for quantitative stereological analysis. Counts were performed similarly for CC1^+^ oligodendrocytes, though a grid size of 150 µm×150 µm and a sampling frame size of 55 µm×55 µm were used. Rostral and caudal counts were summed to give a total number of NeuN^+^ or CC1^+^ cells for each animal. For ED1 staining, the fractionator feature of Stereo Investigator was employed to obtain microglia-macrophage cell counts per area (mm^2^) at 1 mm intervals for up to 2 mm rostral and caudal to the injury epicenter (4 sections per animal). For probe counting, a grid size of 80 µm×80 µm and a sampling frame size of 45 µm×45 µm were used. The counts from each sampled region were added and the Abercrombie equation [48] was applied to obtain estimated numbers of ED1^+^ microglia-macrophages within the 4 mm injured spinal cord segments. The Abercrombie equation estimates total cell number according to the formula: total cell number counted multiplied by the section interval multiplied by the (section thickness/(section thickness/cell diameter)). The average macrophage cell diameter within the injured spinal cord was based upon the previously published value of 13.5+/−0.2 µm [49].

#### Analysis of preserved central myelinated axons

Estimated total numbers of preserved, central myelinated axons (CMAs) were determined by random sampling [50] of peripheral regions of spared white matter surrounding the injury epicenter using computer-assisted microscopy as described previously [39]. In toluidine blue-stained, 1 µm-thick transverse plastic sections (2 sections per animal from a 1 mm spinal cord block encompassing the injury epicenter), the perimeter (contour) of the spared rim of peripheral white matter was contoured and scanned into 50 µm^2^ sized grids using Stereo Investigator. For proper use of this technique, a minimum number of 150–300 myelinated axons were required per white matter area. An estimate of the total number of preserved CMAs was calculated using the formula: (total area within respective contour/total area sampled by fractionator grid)×sum of all axons counted. Preserved CMAs were identified using previously defined characteristics [19]. In the center of the grid, a 10-µm^2^ dissector probe was used for counting preserved CMA profiles. All myelinated profiles within the dissector probe were counted with adjustments for those crossing the border of the probe, and for the thickness of the section. The fractionator probe module allowed for systematic random sampling of CMAs for stereological quantification.

### Behavioral Testing

To examine the functional efficacy of the optimal Rolipram regimen (1.0 mg/kg i.v. at 2 h post-SCI and daily for 2 wk) behavioral testing was performed. Evaluation of gross locomotor performance in the open field was determined according to the BBB scale developed by Basso and colleagues [51]. Testing occurred weekly from 1 to 6 weeks post-injury by two observers blinded to the study group allocation. In addition, a BBB sub-score was employed to provide separate assessment of hindlimb and tail positioning as described elsewhere [52].

### Statistical analysis

A one-way ANOVA followed by a Dunnett post-test was used for comparing counts of CMAs as well as NeuN^+^, CC1^+^ and ED1^+^ cells. Differences from the injury only and vehicle-treated controls were determined independently. Results from the MCP-1 and IL-10 ELISAs, immunoblots or cytokine protein array were statistically compared using a one-way ANOVA and a Tukey post-test. A repeated-measures one-way ANOVA was used for comparison of weekly behavior scores. Differences were accepted to be statistically significant at *p<0.05, **p<0.01 or ***p<0.001 compared to injury only samples, and ^#^p<0.05, ^##^p<0.01 or ^###^p<0.001 compared to vehicle controls. All errors are given as the standard error of the mean.

## Results

### Rolipram exhibits a wide therapeutic dose range for neuroprotection, but a more restricted effective dose profile for oligodendrocyte and CMA sparing

In initial experiments we examined whether acute protection of neurons, oligodendrocytes and CMAs after SCI could be conferred using Rolipram doses ranging from 0.1 mg/kg to 5.0 mg/kg; doses up to 30 mg/kg are known to be well tolerated [53]. For these studies, Rolipram was given by s.c. administration, a route shown previously to be effective in experimental models of SCI [19], [54], first as a single injection at 1 h post-injury and then continued as daily injections for 2 weeks. To assess the protective efficacy of Rolipram following SCI, we performed unbiased stereological analysis of cell and axon counts from coronal, paraffin-embedded or plastic semithin sections (Figure 1) at 2 weeks post-SCI. Counting of NeuN^+^ neurons was performed bilaterally within all laminae; APC-CC1^+^ oligodendrocytes and preserved central myelinated axons were analyzed within the spared, peripheral white matter.

**Figure 2:**
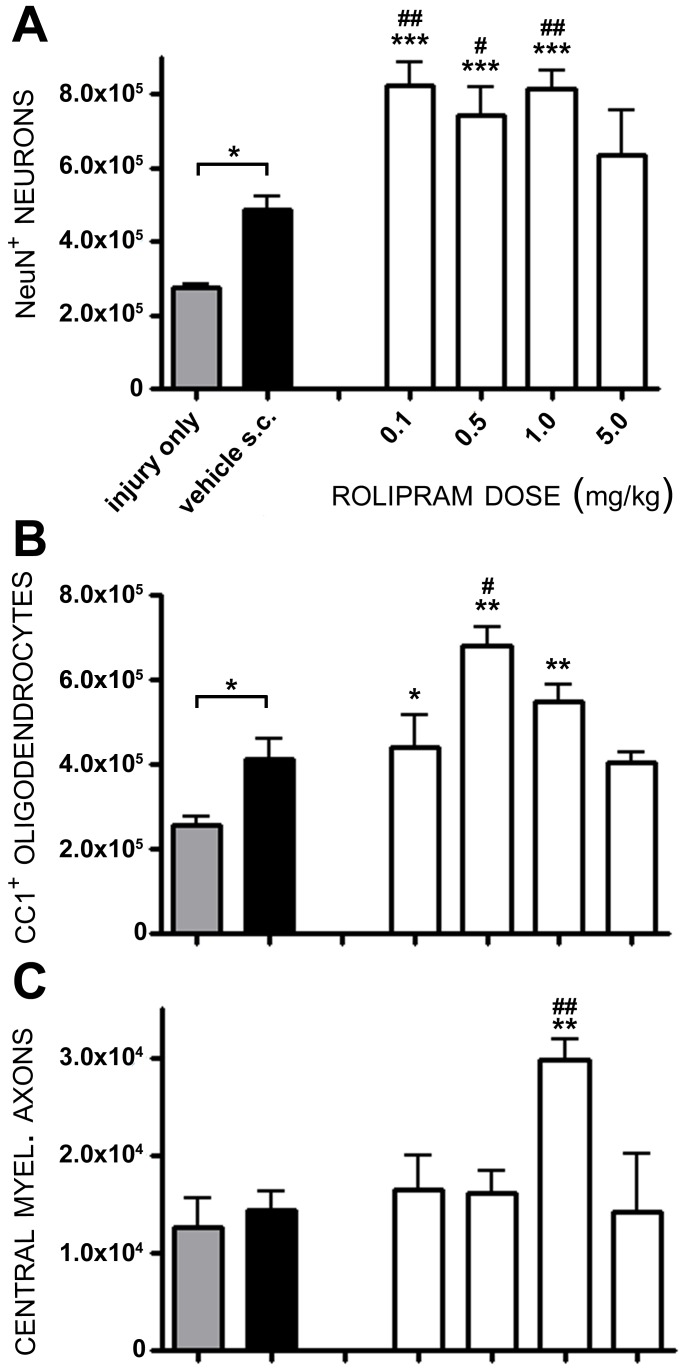
A dose of 1.0 mg/kg of Rolipram provides optimal protection of cells and axons following SCI. Animals were treated with doses of Rolipram ranging from 0.1 mg/kg to 5.0 mg/kg (white bars). Total counts of NeuN^+^ neurons (**A**) or APC-CC1^+^ oligodendrocytes (**B**) within rostral and caudal segments as well as CMAs (**C**) from the injury epicenter, showed that a dose of 1.0 mg/kg Rolipram was optimal for the preservation of axons and cells following SCI. Statistical significance indicated at *p<0.05 or **p<0.01 compared to injury only controls (gray bar) and ^#^p<0.05 or ^##^p<0.01 compared to vehicle controls (black bar). Error bars are expressed as SEMs.

Statistical comparison of numbers of preserved neuronal perikarya within rostral and caudal segments adjacent to the injury epicenter (from 500 to 4,000 µm) showed a significant effect of treatment (F_6,31_ = 15.11, p<0.001) and dose (F_5,26_ = 5.363, p<0.01). Compared to numbers of preserved neurons in injury only controls (2.74×10^5^±1×10^4^ NeuN^+^ neurons), the administration of the vehicle (10% ethanol) alone significantly increased neuronal survival by 78% (4.87×10^5^±3.7×10^4^ NeuN^+^ neurons, *q_31_* = 2.824, p<0.05, Figure 2A). Rolipram delivery at 1 h post-injury, using doses between 0.1 mg/kg and 5.0 mg/kg, also increased numbers of preserved neurons over injury only controls as well as further enhanced neuronal cell preservation compared to vehicle-treated controls, up to a 67% further increase with 1.0 mg/kg (8.13×10^5^±5.2×10^4^ NeuN^+^ neurons, *q_26_* = 3.918, p<0.01, Figure 2A). Rolipram when given at a dose of 5.0 mg/kg, however, did not significantly enhance neuroprotection over vehicle-treated controls (6.37×10^5^±1.2×10^4^ NeuN^+^ neurons, *q_27_* = 1.396, p>0.05).

To assess Rolipram's ability to protect oligodendrocytes following SCI, immunostaining was performed with APC-CC1, a marker for mature oligodendrocytes [43]. Statistical comparison of numbers of preserved oligodendrocytes within rostral and caudal segments adjacent to the injury epicenter (from 500 to 3,000 µm) showed a significant effect of treatment (F_6,31_ = 11.33, p<0.001) and dose (F_5,26_ = 4.785, p<0.05). Compared to numbers of preserved oligodendrocytes within the spared white matter of injury only controls (2.55×10^5^±2×10^4^ CC1^+^ oligodendrocytes, Figure 2B) vehicle delivery alone significantly increased numbers of surviving oligodendrocytes by 61% (4.1×10^5^±5.1×10^4^ CC1^+^ oligodendrocytes, *q_31_* = 2.488, p<0.05). The administration of Rolipram at concentrations of 0.1 to 1.0 mg/kg led to a significant increase in the numbers of preserved oligodendrocytes compared to injury only controls; the highest counts of oligodendrocytes were obtained with 0.5 mg/kg, a 167% increase over injury only controls (6.79×10^5^±4×10^4^ CC1^+^ oligodendrocytes, *q_31_* = 5.727, p<0.01). Rolipram administered at 0.5 mg/kg also enhanced oligodendrocyte preservation a further 66% compared to vehicle-treated controls (*q_26_* = 3.563, p<0.05, Figure 2B). Rolipram, when given at a dose of 5.0 mg/kg, however, did not significantly enhance oligodendrocyte preservation over injury only or vehicle controls (4.03×10^5^±3×10^4^ CC1^+^ oligodendrocytes, *q_31_* = 1.774, p>0.05).

Stereological quantification of spared CMAs within peripheral white matter at the injury epicenter was performed on 1-micron thick plastic sections stained with toluidine blue. Statistical comparison of numbers of spared CMAs at the injury epicenter showed a significant effect of treatment (F_6,31_ = 4.832, *p*<0.01) and dose (F_5,26_ = 5.533, *p*<0.01). Compared to numbers of spared CMAs from injury only controls (1.3×10^4^±3×10^3^ CMAs, Figure 2C) vehicle delivery was without effect (1.4×10^4^±2.1×10^3^ CMAs, *q_32_* = 0.388, p>0.05). Rolipram administration at a dose of 1.0 mg/kg produced significant 131% and 114% increases in the preservation of CMAs over the injury only and vehicle controls, respectively (3.0×10^4^±2×10^3^ CMAs, *q_31_* = 3.800, p<0.01; *q_26_* = 3.551, p<0.01, Figure 2C).

Based on the cyto- and axo-protection results, a Rolipram dose of 1.0 mg/kg was selected as being optimal for continued investigation in subsequent route and therapeutic window studies. This dose consistently provided significant preservation of both cells and axons over vehicle-treated controls (Figure 2A–C).

### The optimal dose of Rolipram provides effective neuroprotection when given via different routes of administration

**Figure 3:**
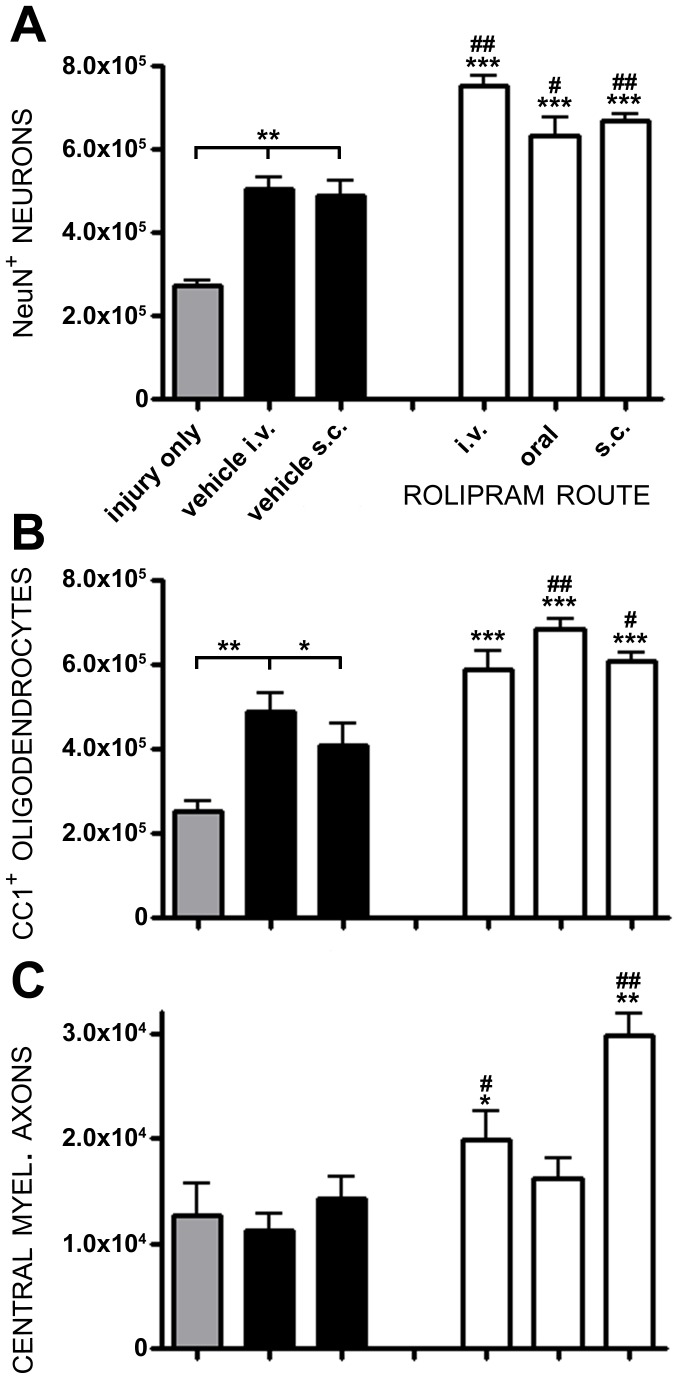
The administration of 1.0 mg/kg Rolipram by intravenous, subcutaneous or oral routes preserves cells and axons post-SCI. All animals received 1.0 mg/kg of Rolipram using i.v., oral or s.c. delivery (white bars). NeuN^+^ neurons (**A**) and AP-CC1^+^ oligodendrocytes (**B**) were significantly protected compared to controls, regardless of the route of Rolipram administration. **C.** CMAs however, were significantly preserved with i.v. and s.c. but not oral, delivery. Statistical significance indicated at **p<0.01 compared to injury only controls (gray bar) and ^#^p<0.05 or ^##^p<0.01 compared to vehicle controls (black bar). Error bars are expressed as SEMs.

Once the optimal dose of Rolipram necessary to provide significant protection of neurons, oligodendrocytes, and CMAs had been defined, experiments to establish the ideal route of delivery were then undertaken. To establish the optimal route, three commonly used approaches were compared: s.c., intravenous i.v. or oral delivery. The histological outcome parameters used to assess cellular and axonal protection (Figure 1) in the initial dose determination study were utilized to discern the optimal route for Rolipram administration. Vehicle (ethanol) delivery demonstrated protective effects on neurons and oligodendrocytes, but not CMAs, when given by either i.v. (neurons, 84% increase, *q*_23_ = 7.488, p<0.01; oligodendrocytes, 93% increase, *q*_23_ = 5.984, p<0.01) or s.c. (neurons, 78% increase, *q*_23_ = 6.963, p<0.01; oligodendrocytes, 61% increase, *q*_23_ = 4.11, p<0.05) routes. No difference in the degree of cytoprotection conferred among routes with ethanol was observed (Figure 3).

Statistical comparison of numbers of preserved neurons showed a significant effect of treatment (F_5,28_ = 39.12, p<0.001) and route (F_4,23_ = 10.92, p<0.001). When compared to SCI only and SCI with vehicle, the administration of 1.0 mg/kg Rolipram, by any of the three administration routes employed, conferred a statistically significant enhancement of neuronal preservation. In comparison to i.v. vehicle controls, there were further 49%, 26% and 32% increases in the numbers of preserved neurons after SCI with intravenous (*q_23_* = 5.596, p<0.01), oral (*q_23_* = 2.938, p<0.05) or subcutaneous (*q_23_* = 3.471, p<0.01) Rolipram administration, respectively (Figure 3A). No significant difference in NeuN^+^ neuron counts was observed between the three administration routes demonstrating that a single route was not preferable to another for mediating neuroprotection (p>0.05).

Statistical comparison of numbers of preserved oligodendrocytes showed a significant effect of treatment (F_5,28_ = 27.02, p<0.001) and route (F_4,23_ = 6.772, p<0.01). Oligo-protection was provided by either oral (a 40% increase, *q_23_* = 4.484, p<0.01) or subcutaneous Rolipram delivery (a 24% increase, *q*_23_ = 2.734, p<0.05), but not intravenous (*q*_23_ = 2.049, p>0.05) as compared to SCI with vehicle (Figure 3B). However, neither route produced a significant increase in oligodendrocyte preservation over the other. Statistical comparison of numbers of preserved CMAs showed a significant effect of treatment (F_5,28_ = 11.41, p<0.001) and route (F_4,23_ = 16.49, p<0.001). With CMA preservation, while vehicle delivery was not effective in conferring protection compared to SCI only controls, 1.0 mg/kg Rolipram when given intravenously (a 77% increase *q_23_* = 2.719, p<0.05) or subcutaneously (a 166% increase, *q_23_* = 6.908, p<0.01), but not orally (*q*_23_ = 2.124, p>0.05), increased numbers of CMAs within the peripheral white matter (Figure 3C).

Although all routes of administration allowed Rolipram to confer some degree of cellular or axonal protection, the most effective methods were intravenous and subcutaneous. Based upon clinical feasibility, in which the most rapid delivery method would be via an intravenous line that would have already been placed in all persons with SCI, we chose to pursue further therapeutic window evaluation studies with the daily intravenous administration of 1.0 mg/kg Rolipram for a period of 2 weeks.

### The therapeutic window for Rolipram as an acute protective agent after SCI is of long duration and is cell specific

In light of the difficulties associated with the timing of an acutely administered and experimental therapeutic modality to SCI individuals under clinical trial conditions it is important to identify the effective therapeutic window of a neuroprotectant to ensure that it can be translated successfully to the clinic. Based upon previous clinical trials of pharmacological agents in SCI, such as methylprednisolone [55] and GM-1 ganglioside [56] it is very difficult to administer a pharmacological agent to a person with SCI under experimental Phase I/II conditions within 3 hours of injury due to the need for hospital transfer, treatment of concomitant complications and obtaining informed consents from the SCI person or family members [57]. Therefore, although in the first parts of this investigation Rolipram provided profound cellular and axonal protection within 1 hour of injury, a longer therapeutic window would be more favorable for experimental investigation of its clinical utility. Therefore in these studies we determined the therapeutic window of Rolipram for cyto- and axo-protection when administered between 2 h and 48 h after SCI using the previously established optimal regimen, intravenous delivery at 1.0 mg/kg for 2 weeks post-injury.

**Figure 4:**
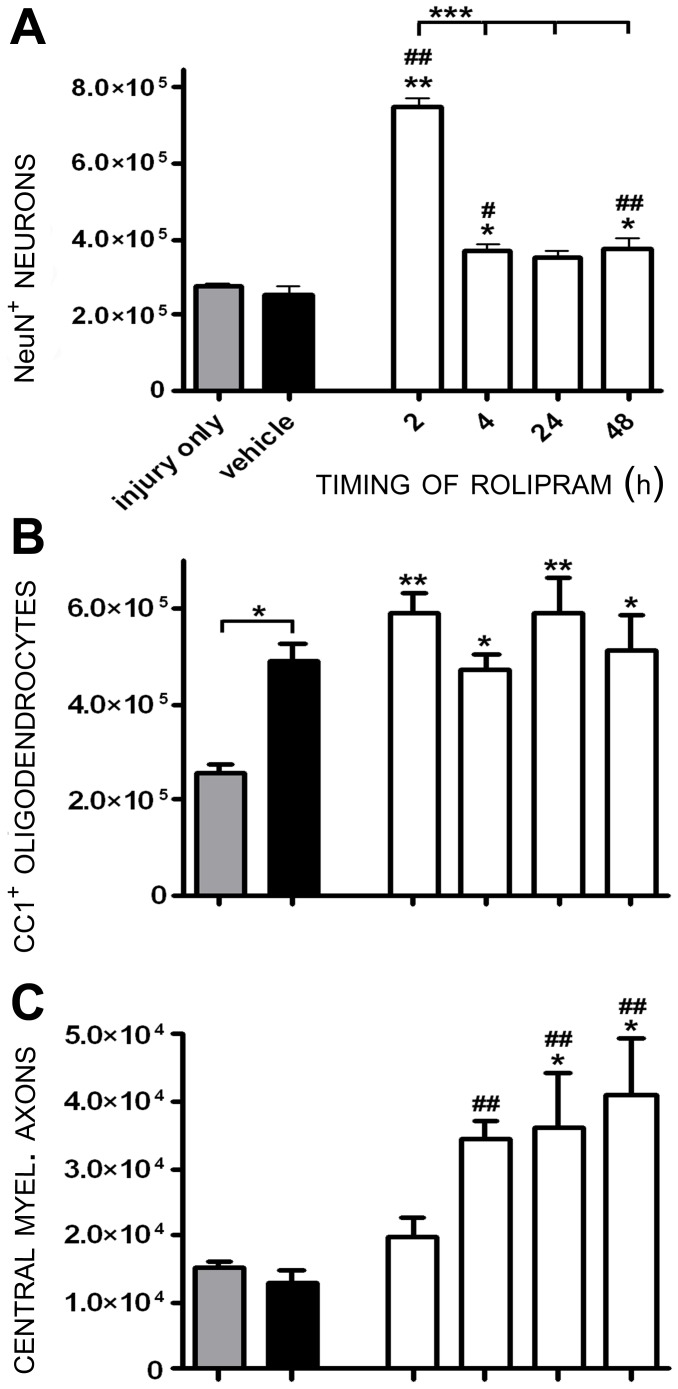
Rolipram administration initiated between 2 and 48 h post-injury enhances neuron survival and provides axo-protection. Rolipram (1.0 mg/kg) was administered i.v. starting at specific times (1–48 h) post-injury (white bars). **A.** NeuN^+^ neurons were significantly protected when Rolipram was administered between 2 and 48 h post-injury, though 2 h post-injury was the most effective initial delivery time (showed significantly greater NeuN^+^ neurons compared to Rolipram given at later times, ***p<0.001). **B.** No significant preservation of oligodendrocytes was observed with any of the delivery times employed when Rolipram was used intravenously compared to vehicle controls. **C.** CMA preservation was achieved when i.v Rolipram was initiated up to 48 h post-SCI. Statistical significance indicated at *p<0.05 or **p<0.01 compared to injury only controls (gray bar) and ^#^p<0.05 or ^##^p<0.01 compared to vehicle controls (black bar). Error bars are expressed as SEMs.

Statistical comparison of numbers of preserved neurons showed a significant effect of treatment (F_7,43_ = 57.28, p<0.001) and administration time (F_5,29_ = 60.58, p<0.001). Rolipram provided marked 174% and 196% increases in neuronal preservation compared to SCI only (*q_43_* = 14.88, p<0.01) and SCI with vehicle (*q_38_* = 14.58, p<0.01) controls, respectively, when delivered at 2 h post-injury (Figure 4A). Significant neuron protection versus vehicle controls, albeit to a lesser extent than 2 h, was also observed when Rolipram administration was initiated at later times following SCI; increases of 45% at 4 h (*q_38_* = 3.370, p<0.05), 38% at 24 h (*q_38_* = 2.799, p<0.05) and 47% at 48 h (*q_38_* = 3.479, p<0.01) post-injury were observed in numbers of NeuN^+^ neurons compared to vehicle controls (Figure 4A). A statistically significant elevation in neuronal preservation with Rolipram at 2 h compared to later administration delays (p<0.001 at all time-points) clearly demonstrated the benefit of a rapid administration of Rolipram for maximal neuroprotective efficacy.

Statistical comparison of numbers of preserved oligodendrocytes showed a significant effect of treatment (F_7,43_ = 3.753, p<0.01) but not administration time (F_5,29_ = 0.9469, p>0.05). Stereological quantification of numbers of preserved CC1^+^ oligodendrocytes revealed that Rolipram administration, when started 2 h to 48 h post-injury, significantly increased numbers of preserved oligodendrocytes compared to injury only controls (up to 132% at 2 h and 24 h, *q_38_* = 3.96, p<0.01; *q_38_* = 4.19, p<0.01, Figure 4B); however, these changes were not significant to the preservation afforded by vehicle administration, substantiating the findings obtained with ethanol-mediated oligo-protection in the earlier parts of this investigation.

Statistical comparison of numbers of preserved CMAs showed a significant effect of treatment (F_7,43_ = 5.617, p<0.001) but not administration time (F_5,29_ = 1.803, p>0.05). Rolipram administration initiated 4 h through 48 h post-SCI produced significant protection of CMAs compared to SCI only and/or SCI with vehicle controls; increases in CMA counts compared to vehicle were 170% at 4 h (*q_38_* = 3.555, p<0.01), 182% at 24 h (*q_38_* = 3.814, p<0.01) and 221% at 48 h (*q_38_* = 4.053, p<0.01). No statistically significant difference in the degree of CMA preservation, however, was observed between these timepoints (Figure 4C).

**Figure 5:**
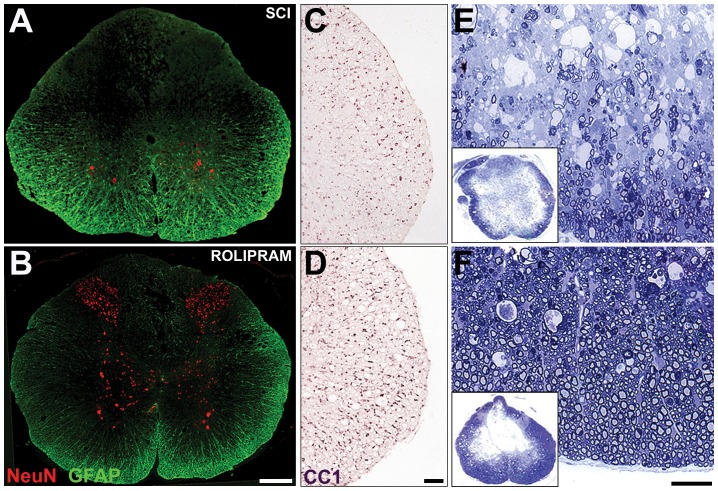
The optimal administration protocol of Rolipram provides significant neuro- and axo-protection. Representative images from animals that received the optimal Rolipram administration protocol, 1.0 mg/kg given i.v. at 2 h post-injury (bottom row), or injury only controls (top row). **A–B.** In sections 2 mm caudal to the lesion, significantly more NeuN^+^ neurons are preserved in all gray matter laminae in Rolipram (**B**) versus injury only controls (**A**). Sections were co-stained with GFAP to reveal the spinal cord architecture. **C–D.** In sections 2 mm caudal to the lesion, significantly more APC-CC1^+^ oligodendrocytes are identified in the lateral white matter of Rolipram treated (**D**) versus injury only controls (**C**). However, this degree of preservation was also observed in those animals receiving ethanol (vehicle). **E–F.** In toluidine blue-stained 1 µm semithin sections from the injury epicenter, more preserved CMAs were found in the spared peripheral white matter in animals receiving Rolipram (**F**) compared to injury only controls (**E**).

In sum, these findings demonstrate that although Rolipram administration within 2 h following SCI is the optimal therapeutic window for the provision of neuroprotection, this window is significantly extended for Rolipram's action in limiting axon demyelination; persistent efficacy is seen for up to 48 h post-SCI. These results point to the difference in timing of these pathological processes and thus their optimal therapeutic windows for a protective pharmacological regimen. The comparative protective efficacy of the optimal Rolipram protocol (2 h post-injury, intravenous at 1.0 mg/kg) can be appreciated by contrasting images of preserved neurons (Figure 5A–B), oligodendrocytes (Figure 5C–D) and CMAs (Figure 5E–F) to injury only controls.

### The optimal neuroprotective regimen for Rolipram improves locomotion after SCI

**Figure 6:**
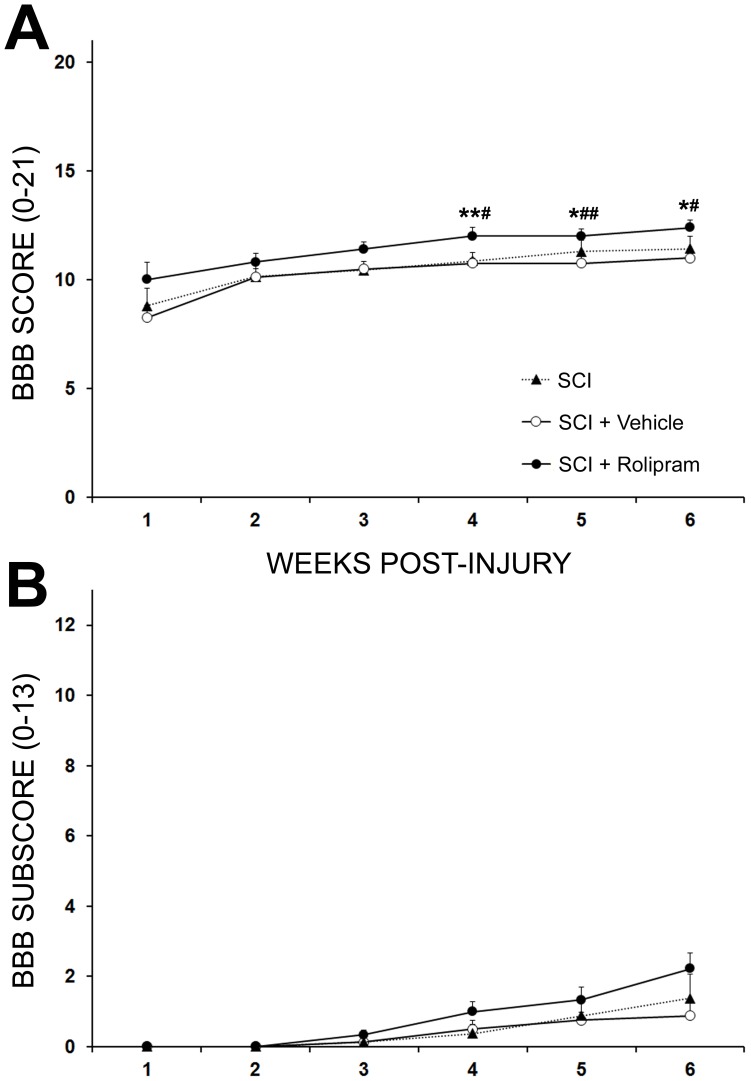
The optimal regimen for Rolipram delivery improves functional outcome after SCI. **A.** Locomotor function was evaluated weekly in the open-field using the BBB score. Rolipram treated animals (black circle) exhibited a trend for higher BBB scores from week 1 post-SCI, with statistically significant improvements over SCI only (*, black triangle) or SCI with vehicle (^#^, white circle) controls from week 4 post-SCI to endpoint. **B.** Although Rolipram-treated animals exhibited the highest average BBB subscore at endpoint, no significant differences among groups were found post-SCI. Statistical significance indicated at *p<0.05 or **p<0.01 compared to injury only controls and ^#^p<0.05 or ^##^p<0.01 compared to vehicle controls. Error bars are expressed as SEMs.

To assess the effectiveness of the optimal Rolipram administration protocol to improve functional recovery after SCI, gross locomotor performance in the open field was assessed weekly using BBB score and subscore analysis. Statistical comparison of BBB scores showed a significant effect of treatment at endpoint (F_3,24_ = 6.280, p<0.01). Although no significant difference in locomotor performance was observed between controls, SCI alone or SCI with vehicle, and Rolipram-treated animals during the first 3 weeks post-injury (3 week BBB scores: 10.3±0.4 or 10.5±0.3 vs. 11.3±0.2, respectively; *q_24_* = 3.535 and 2.719, respectively; p>0.05; Figure 6A), a statistically significant improvement was seen from 4 weeks post-SCI to endpoint. At endpoint, animals receiving Rolipram performed significantly better in the open field than the controls (6 week BBB scores: 12.9±0.4, Rolipram vs. 11.1±0.6, SCI, *q_24_* = 4.144, p<0.05 and 11.0±0.3, Vehicle, *q_24_* = 4.438, p<0.05; Figure 6A). No benefit was provided by vehicle administration over the SCI only control. BBB subscore analysis revealed no statistically significant differences between groups during the post-injury evaluation period, with BBB subscores at endpoint for SCI only, vehicle and Rolipram groups being 1.4±0.7, 0.9±0.4 and 2.2±0.5, respectively (Figure 6B).

### The predominant PDE4 target for Rolipram's neuroprotective actions are PDE4B1 and PDE4A5; PDE4A is highly expressed and phosphorylated in neurons and glia after SCI

In order to identify the PDE4 target of Rolipram's neuroprotective action following SCI, we first performed immunoblot analysis for protein levels and the phosphorylation status of PDE4A, B and/or D (PDE4C is not expressed in the CNS; an antibody that specifically recognizes pPDE4B is not available) at 2, 4 and 25 h post-SCI in injury only, vehicle and Rolipram-treated (1 h or 24 h, i.v.) animals. The primary mechanism of action by which Rolipram reduces cyclic AMP hydrolysis is to bind PDE4 and prevent its phosphorylation and ensuing hydrolytic activity [24], which then putatively confers cytoprotective effects [19], [58].

**Figure 7:**
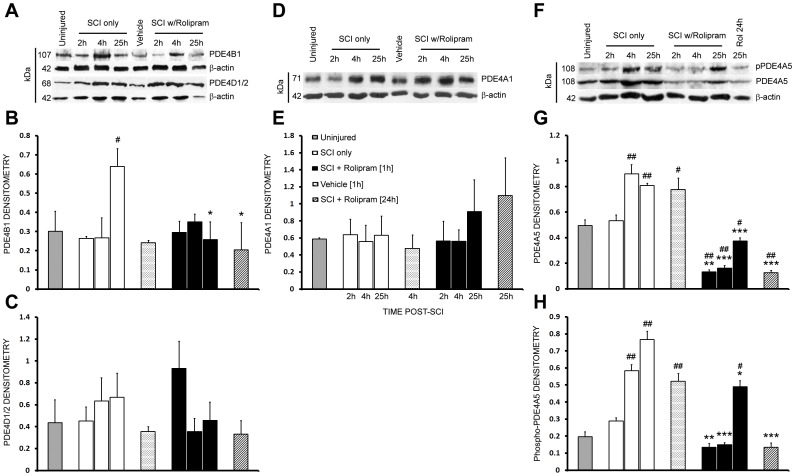
Rolipram reduces SCI-induced increases in PDE4B1 and PDE4A5 protein production as well as PDE4A5 phosphorylation. **A–C.** Immunoblot analysis of injured spinal cord homogenates revealed an SCI-induced increase in PDE4B1 at 25 h that was significantly abrogated by Rolipram treatment, when given at either 1 or 24 h after SCI (**A, B**). PDE4D1/2 was not regulated by either SCI or Rolipram treatment at the protein level (**A, C**). **D–H.** Unlike PDE4A1 (**D–E**), PDE4A5 protein production (**F, G**) and phosphorylation (**F, H**) was increased from 4 to 25 h after SCI. Rolipram, when given at either 1 or 24 h after SCI (black and diagonal line bars, respectively), significantly abated these SCI-induced increases. Vehicle delivery (at 1 h, dotted white bars) did not affect the protein production or phosphorylation of the PDE4s examined post-SCI. Density readings of PDE4 bands were normalized to β-actin immunoreactivity performed on the same blots. Statistical significance indicated at *p<0.05, **p<0.01 or ***p<0.001 compared to injury only controls (white bars) and ^#^p<0.05 or ^##^p<0.01 compared to uninjured controls (gray bars). Error bars are expressed as SEMs.

Immunoblot detection of PDE4B with a pan-PDE4B antibody revealed a band at ∼107 kDa (PDE4B1) that was temporally regulated following SCI (F_4,16_ = 4.368, p<0.05; Figure 7A–B). Although no significant change in PDE4B1 was observed at 2 and 4 h post-injury, or with vehicle or Rolipram at those times, PDE4B1 was increased 212% at 25 h after SCI (*q_16_* = 3.145, p<0.05). The administration of Rolipram, at either 1 or 24 h after SCI, abated this increase in PDE4B1 expression at 25 h post-injury, bringing PDEB1 expression to levels comparable to the uninjured controls (two-tailed t test; *t_9_* = 2.916, p<0.05 and *t_9_* = 2.754, p<0.05, respectively). Examination of PDE4D with a pan-PDE4D antibody detected a band at 68 kDa (PDE4D1 or 2, same MW); PDE4D1/2 was not regulated after SCI or when either vehicle or Rolipram was administered (Injury; F_4,16_ = 0.3815, p>0.05; Figure 7A, C).

The use of a pan-PDE4A antibody resulted in the identification of two bands, one at 71 kDa, which was not regulated after SCI, PDE4A1 (F_4,16_ = 0.048, p<0.05; Figure 7D–E), or following vehicle or Rolipram administration. The second band at 108 kDa, PDE4A5, was temporally regulated post-injury (F_4,16_ = 16.5, p<0.001; Figure 7F–G). Although no significant change in PDE4A5 expression was observed at 2 h post-SCI, increases of 182% (*q_16_* = 5.79, p<0.01) and 163% (*q_16_* = 4.495, p<0.01) were observed at 4 and 25 h, respectively. Rolipram administration at 1 h significantly reduced PDE4A5 expression at 2 h (*t_9_* = 8.284, p<0.01), 4 h (*t_9_* = 9.71, p<0.001) and 25 h (*t_9_* = 14.219, p<0.001) post-SCI, to levels that were significantly lower than that of uninjured controls (2 h, *q_16_* = 9.097, p<0.01; 4 h, *q_16_* = 8.342, p<0.01; 25 h, *q_16_* = 3.017, p<0.05). Rolipram administration at 24 h after SCI also abated PDE4A5 expression at 25 h (*t_9_* = 26.73, p<0.001), to levels significantly lower than that of uninjured controls (*t_9_* = 7.746, p<0.01). Vehicle was without effect on SCI-induced PDE4A5 expression.

To evaluate phosphorylated PDE4A, we performed immunoblotting using a pan-PDE4A antibody that recognizes serine-phosphorylated PDE4A long-forms. We identified the phosphorylated form of PDE4A5 (108 kDa), which increased following SCI (Figure 7F, H). There was no change in pPDE4A5 at 2 h after SCI (*q_16_* = 1.897, p>0.05) but significant increases of 296% (*q_16_* = 8.028, p<0.01) and 389% (*q_16_* = 11.81, p<0.01) occurred at 4 and 25 h, respectively (Figure 7H). Rolipram prevented the SCI-induced increase in pPDE4A5 at all timepoints compared to injury only controls (2 h, *t_9_* = 5.467, p<0.01; 4 h, *t_9_* = 12.01, p<0.001; 25 h, *t_9_* = 4.571, p<0.05; Figure 7I). When Rolipram was administered at 24 h, at 25 h post-SCI a significant reduction in pPDE4A5 (*t_9_* = 11.46, p<0.001) was observed (Figure 7F, H). Vehicle administration did not significantly affect the level of phosphorylated PDE4A5 compared to SCI only controls.

**Figure 8:**
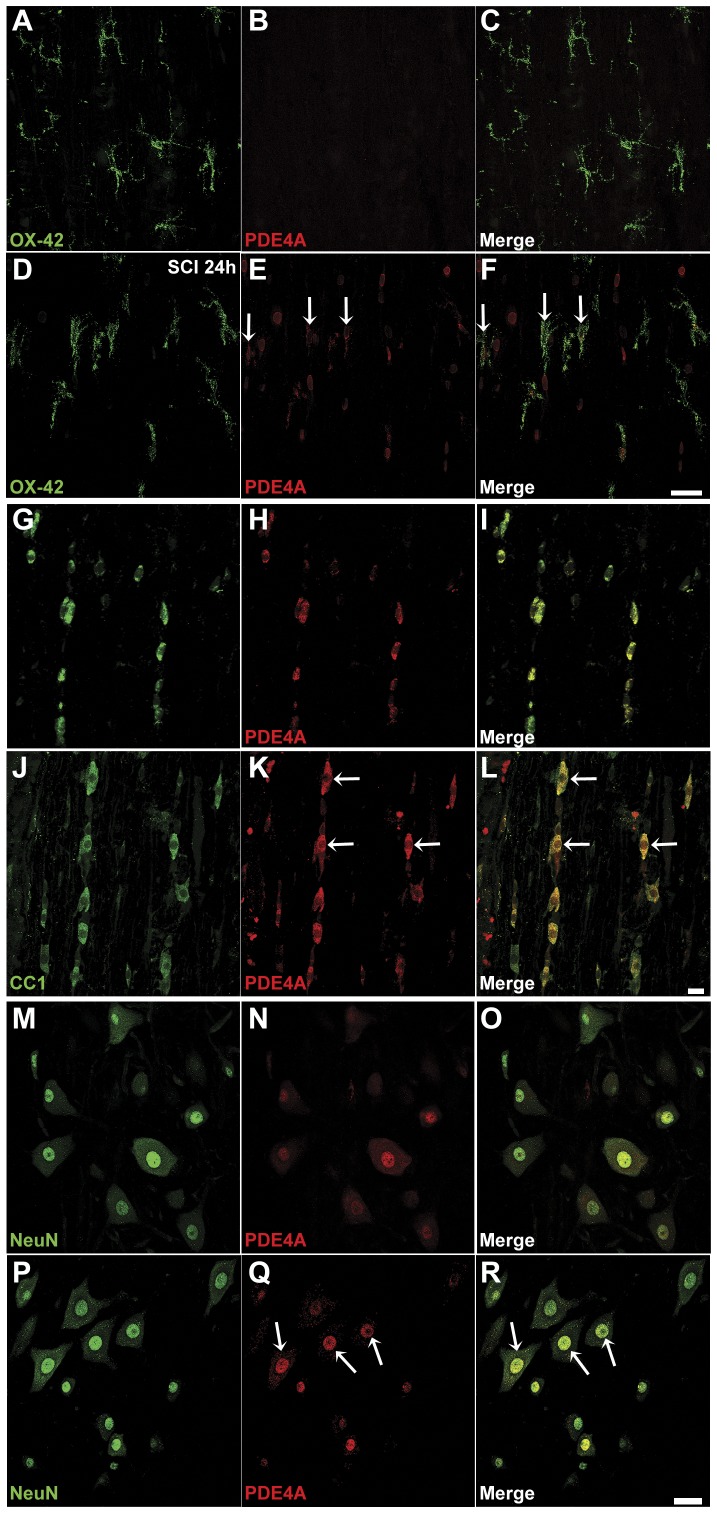
Immunochemical detection of PDE4A protein at 24 h after SCI shows an induction of expression in microglia, but no apparent change in neurons or oligodendrocytes. **A–C.** PDE4A (red) is virtually absent from OX-42^+^ microglia (green) in uninjured spinal cord. **D–F.** At 24 h post-SCI there is an induction of PDE4A in microglia, with localization to the cytoplasm and processes. **G–L.** APC-CC1^+^ oligodendrocytes within uninjured spinal cord show PDE4A expression throughout the cell body (**G–I**) that appears unchanged at 24 h post-SCI (**J–L**). **M–R.** Similarly, basal PDE4A expression in NeuN^+^ neurons (**M–O**), which appears cytoplasmic, peri-nuclear and/or nuclear, appears unchanged at 24 h post-SCI (**P–R**). White arrows indicate cells that have immunochemical co-localization of cell-specific markers and PDE4A. Scale bars = 40 µm.

**Figure 9:**
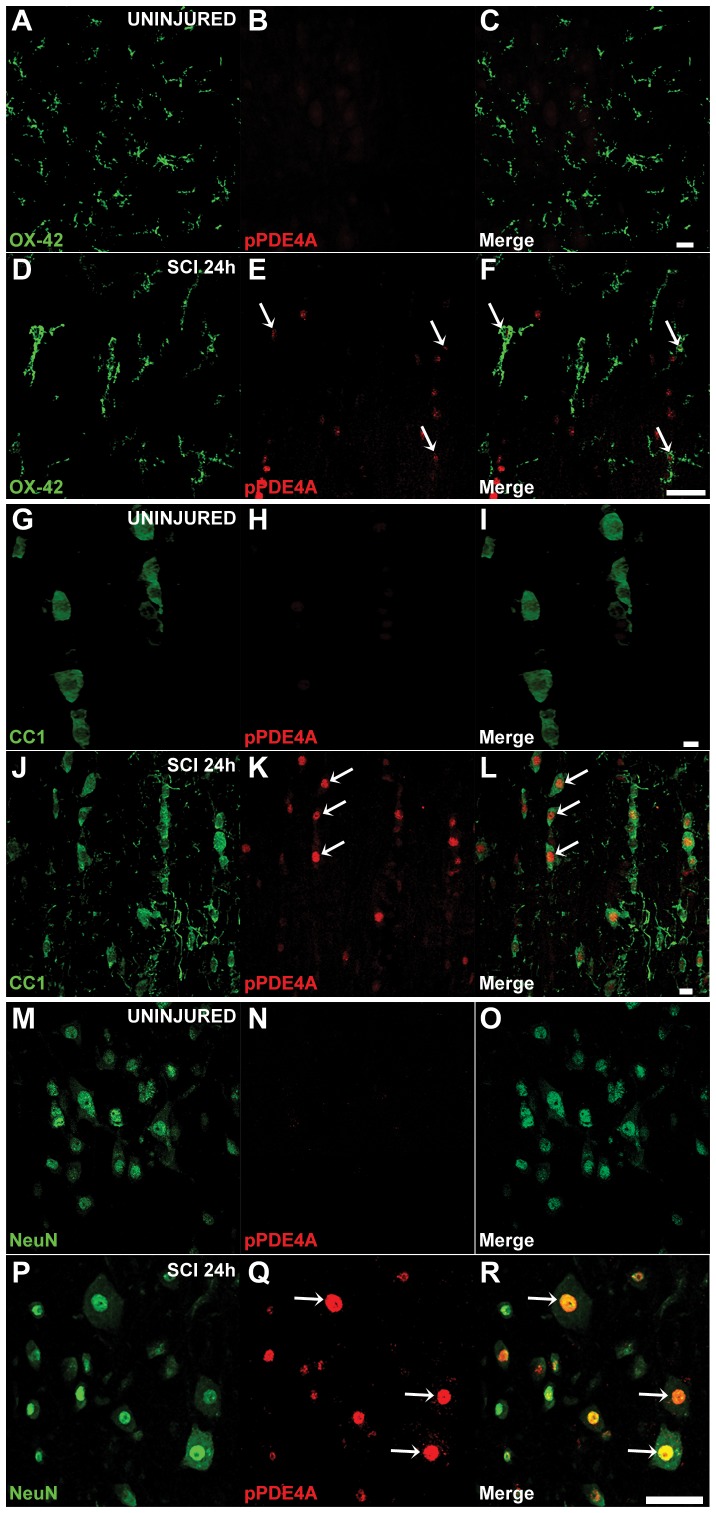
Immunochemical detection of PDE4A phosphorylation at 24 h after SCI shows strong induction in microglia, neurons and oligodendrocytes. Barely detectable PDE4A phosphorylation (red) was observed in microglia (**A–C**), oligodendrocytes (**G–I**) and neurons (**M–O**) within the uninjured spinal cord. At 24 h following SCI, pronounced PDE4A phosphorylation was detected in all cell types: microglia (**D–F**), oligodendrocytes (**J–L**) and neurons (**P–R**). White arrows indicate cells that have immunochemical co-localization of cell-specific markers and phosphorylated PDE4A. Scale bars = 40 µm.

To identify the cellular location of PDE4A and pPDE4A in uninjured spinal cord tissue and at 24 h post-SCI, we next performed immunohistochemistry with pan-PDE4A (Figure 8) and phosphorylated-PDE4A-specific (Figure 9) antibodies in combination with the cell-specific markers; OX-42 (microglia-macrophages), CC1 (oligodendrocytes) and NeuN (neurons). Confocal images taken from immediately rostral to the injury site at 24 h post-SCI or from an analogous region in uninjured controls showed that oligodendrocytes (Figure 8G–I) and neurons (Figure 8M–O) expressed PDE4A, but not microglia (Figure 8A–C), in the uninjured spinal cord. Microglia (Figure 8D–F), oligodendrocytes (Figure 8J–L) and neurons (large motor neurons shown; Figure 8P–R), however, all expressed significant PDE4A after SCI. PDE4A appeared localized to the peri-membrane, cytoplasmic, peri-nuclear and nuclear regions. In comparison, phosphorylated PDE4A, which was largely absent in all these cell types within uninjured controls, was found at 24 h post-SCI in microglia (Figure 9A–F), oligodendrocytes (Figure 9G–L) and neurons (Figure 9M–R), with its localization being predominantly nuclear but also cytoplasmic.

### Rolipram administration produces temporal changes in the production of specific pro-inflammatory and suppressor cytokines following SCI

**Figure 10:**
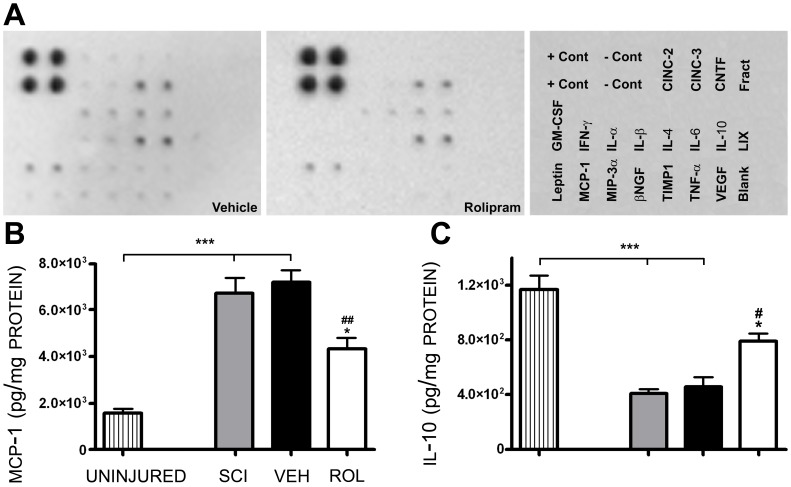
Acute Rolipram treatment reduces monocyte chemoattractant protein levels while delayed administration increases interleukin-10 levels. Spinal cord encompassing the injury epicenter or the uninjured T8 segment was harvested and analyzed for concentrations of cytokines by protein array (See Table 2) or used to measure MCP-1 and IL-10 using specific ELISAs. **A.** Representative images from cytokine protein arrays of SCI with vehicle and SCI with Rolipram (at 1 h post-SCI) animals 2 h post-injury. The far right panel shows the arrangement of cytokine antibodies and positive/negative controls on the membrane. **B.** ELISA shows a significant increase in MCP-1 at 2 h post-SCI compared to uninjured controls (lined bar) that was attenuated by Rolipram (white bar), but not vehicle, when given at 1 h. **C.** IL-10 protein levels were significantly reduced at 25 h post-SCI compared to uninjured controls (lined bar). Rolipram (white bar), but not vehicle, partially prevented the SCI-induced decrease in IL-10 when given at 24 h. Statistical significance indicated at *p<0.05 compared to injury only controls (gray bars) and ^#^p<0.05 or ^##^p<0.01 compared to vehicle controls (black bars). Error bars are expressed as SEMs.

To identify the mechanism(s) responsible for the protective actions of Rolipram, we performed a protein cytokine dot blot array to evaluate immunomodulatory changes in the production of 19 cytokines and chemokines at two timepoints post-SCI, vehicle and/or Rolipram treatment, 2 h and 25 h (Rolipram or vehicle was given 1 h prior to these endpoints). Spinal cord encompassing the injury epicenter was collected and assayed. Evaluation of protein production 2 h post-SCI using this method firstly identified a number of cytokines that were up-regulated (GM-CSF, IL-6, IL-10 and β-NGF) or down-regulated (IL-4, MIP3α, TIMP-1 and TNF-α) with vehicle administration at 1 h (Table 2; Figure 10A). Comparative to vehicle, Rolipram further increased the production of GM-CSF, IL-6 and IL-10, reduced to a greater extent MIP3α and additionally down-regulated IL-1α and IL-1β. In contrast to vehicle, Rolipram up-regulated the production of IL-4 and TIMP-1. At 25 h after SCI, when Rolipram was administered at either 1 h or 24 h post-injury, a similar pattern of regulation of cytokine production was observed to the 2 h timepoint, albeit to a lesser extent.

**Table 2:** Cytokines altered by Rolipram administration after spinal cord injury.

	2 hours after SCI		25 hours after SCI	
Cytokine	Vehicle*(1)	Rolipram*(1)	Rolipram*(2)	Rolipram#(2)
CINC-2	No change	No change	No change	No change
CINC-3	No change	No change	No change	No change
GM-CSF	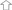 1.8 fold	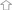 9.6 fold	No change	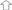 1.6 fold
IL-1α	No change	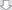 1.8 fold	No change	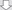 3.0 fold
IL-1β	No change	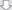 3.0 fold	No change	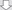 3.0 fold
IL-4	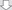 1.4 fold	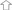 2.3 fold	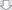 2.0 fold	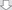 5.0 fold
IL-6	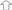 1.3 fold	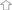 2.1 fold	No change	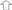 1.8 fold
IL-10	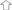 1.9 fold	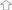 4.3 fold	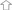 4.4 fold	** 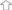 3.0 fold**
Leptin	No change	No change	No change	No change
MCP-1	No change	** 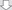 5.0 fold**	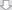 2.5 fold	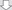 4.0 fold
MIP-3α	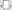 2.0 fold	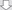 3.0 fold	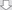 1.8 fold	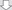 4.0 fold
β-NGF	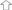 1.8 fold	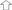 2.0 fold	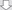 1.8 fold	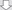 1.8 fold
TIMP-1	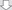 1.6 fold	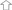 4.2 fold	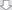 2.0 fold	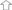 2.3 fold
TNF-α	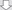 4.0 fold	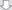 4.0 fold	No change	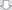 5.5 fold

A cytokine protein array (RayBiotech Inc.) was used to identify changes in the production of 19 cytokines after SCI and/or vehicle or Rolipram administration. Fold-changes, based upon densitometry readings (normalized across arrays by positive and negative controls), indicate changes with Rolipram or vehicle treatment versus SCI controls. Symbols are as follows:

(*) delivered at 1 hour post-injury;

(#) delivered at 24 hours post-injury; (1) change compared to SCI control at 2 hours; (2) change compared to vehicle control at 25 hours. The changes (increase/decrease) indicated by the bolded font were subsequently confirmed using Ab-specific ELISA. Note, the following cytokines were not detected under any conditions and time points on the assay: CNTF, Fracktalkine, IFN-γ, LIX and VEGF.

From this initial screen, we identified two cytokines (one from each administration paradigm of 1 and 24 h post-SCI) that exhibited a significant change in response to Rolipram treatment as compared to SCI and vehicle controls for additional confirmatory analysis using ELISA. In the acute paradigm, Rolipram administration significantly reduced levels of the monocyte/T cell chemokine [58], Monocyte Chemoattractant Protein-1 (MCP-1; Table 2), while in the delayed paradigm, Rolipram increased production of the suppressor cytokine and neuroprotective protein [59], interleukin-10 (IL-10; Table 2). Using samples of similarly-treated animals to the cytokine array, we next performed ELISAs specifically for MCP-1 and IL-10. Compared to uninjured controls (1,500±190 pg/mg protein), MCP-1 protein levels were dramatically increased 335% at 2 h post-SCI (6,700±630 pg/mg protein; *t_12_* = 10.20, p<0.001; Fig. 10B). Levels of MCP-1 were unaffected by vehicle administration but were significantly reduced 40% by Rolipram (4,300±480 pg/mg protein; *t_12_* = 6.066, p<0.01) when it was given 1 h prior, compared to SCI only controls. MCP-1 levels though remained elevated 179% compared to uninjured control levels after SCI and Rolipram (*t_12_* = 5.082, p<0.05; Figure 10B). In comparison to uninjured controls (1,200±110 pg/mg protein), IL-10 protein levels were significantly reduced 66% at 25 h post-SCI (410±29 pg/mg, *t_12_* = 8.869, p<0.001; Figure 10C). Rolipram, but not vehicle delivery, at 24 h post-SCI significantly attenuated this reduction in IL-10 by 42% (790±53 mg/pg protein, *t_12_* = 4.292, p<0.05) compared to the 25 h injury only control (458±69 mg/pg protein; Figure 10C).

### Rolipram reduces mononuclear phagocyte infiltration/activation within the injured spinal cord

**Figure 11:**
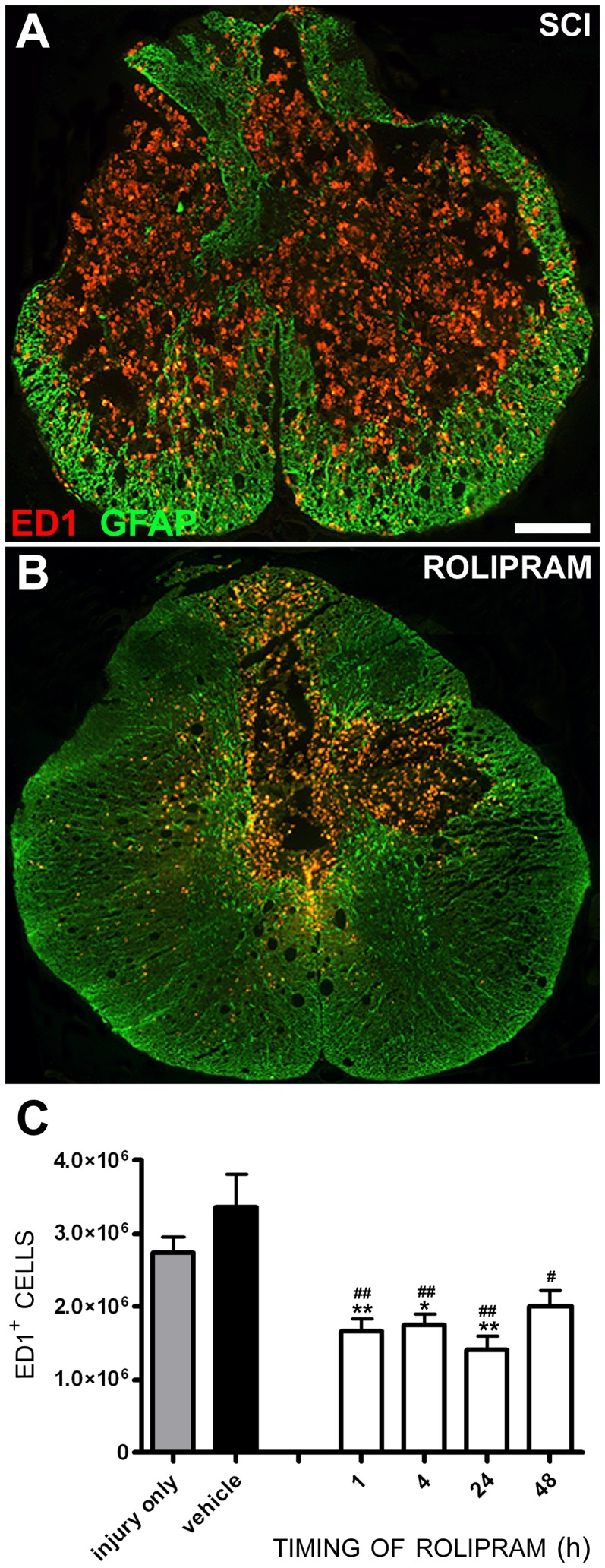
Rolipram treatment reduces the number of ED1-positive monocytic phagocytes within the injured spinal cord. **A–C.** Rolipram (1.0 mg/kg, i.v.), when administered within 24 h post-injury reduced the number of ED1^+^ macrophages within the injured spinal cord compared to SCI only and SCI vehicle-treated controls (**C**). Coronal sections from 2 mm caudal to the injury epicenter of SCI only (**A**) and SCI with Rolipram (**B**, given at 1 h) animals shows a dramatic reduction of ED1^+^ macrophages-microglia within the injured spinal cord provided by Rolipram when assessed 2 weeks post-SCI. Sections are co-stained with GFAP to provide the architecture of the spinal cord. Scale bar = 400 µm. Statistical significance indicated at *p<0.05 or **p<0.01 compared to injury only controls (gray bar) and ^#^p<0.05 or ^##^p<0.01 compared to vehicle controls (black bar). Error bars are expressed as SEMs.

The importance of MCP-1 and IL-10 in regulating immune cell infiltration, migration and activation following tissue injury would imply that by altering their production, Rolipram may have modulated the immune response in a negative fashion at one or more of these stages to mediate cytoprotection. To determine if Rolipram had altered immune cell infiltration/activation, we performed staining for the lysosomal protein recognized by the antibody ED1 in activated mononuclear phagocytes such as macrophages and microglia [42], to quantify their numbers within a 5 mm segment of spinal cord encompassing the injury epicenter in SCI only, vehicle and Rolipram-treated animals at 2 weeks post-SCI. For these studies Rolipram was given starting at 1, 4, 24 or 48 h post-injury. Compared to SCI only controls (2.73×10^6^ ED1^+^ cells; Figure 11A, C), vehicle delivery had no effect on numbers of ED1^+^ cells within the lesion (3.35×10^6^ ED1^+^ cells, *t_17_* = 1.729, p>0.05; Figure 11C). In contrast, Rolipram delivery via the optimal dose and route at 1–48 h post-injury, significantly attenuated the numbers of ED1^+^ macrophages and microglia within the injured cord compared to SCI only controls at all timepoints except 48 h (Figure 11C); a 39% decrease in ED1^+^ cells was observed when Rolipram was given starting at 1 h (1.67×10^6^±2.7×10^5^ cells, *t_14_* = 3.915, p<0.01; Figure 11B, C), a 36% decrease starting at 4 h (1.74×10^6^±2.58×10^5^, *t_14_* = 3.654, p<0.05), and a 49% decrease starting at 24 h (1.4×10^6^±3.36×10^5^, *t_14_* = 4.916, p<0.01). No significant differences in ED1^+^ cell numbers were observed between Rolipram administration cohorts.

## Discussion

Previously we and others have demonstrated that cyclic AMP elevation, through administration of the PDE4 inhibitor Rolipram, can provide neuroprotection following CNS injury [19], [20], [60]–[62]. The translation of Rolipram towards clinical evaluation as a potential therapy for limiting secondary damage and reducing the degree of functional deficit after SCI, however, required optimization of the drug's dosing and delivery route as well as the establishment of its therapeutic window. To provide putative biomarkers of drug efficacy and data for the development of the next generation of PDE4 inhibitors for CNS protection, the identification of the PDE4 protein target(s) of Rolipram in the injured CNS as well as an improved understanding of its mechanism of action as a neuroprotectant was also needed.

In the current study the dose-delivery protocol for Rolipram as a neuroprotectant was optimized. An effective daily dose between 0.1 and 1.0 mg/kg was identified, well within the tolerable dose of 30 mg/kg in rats [53], and a preferred method of delivery, via the intravenous route with daily dosing for 2 weeks post-injury, provided the greatest efficacy. Evaluation of the temporal therapeutic window of Rolipram for white and gray matter preservation after contusive SCI demonstrated that the drug was effective, particularly for white matter preservation, even when first administered at 48 hours post-injury. Importantly, the most effective administration protocol for Rolipram provided tissue preservation that was functionally meaningful, with significant improvements after SCI in open-field locomotion as measured using the BBB score. Using the optimal delivery protocol for Rolipram, reductions in PDE4B1 and PDE4A5 protein production were observed as well as a decrease in the serine phosphorylation of PDE4A5; serine phosphorylation within the upstream conserved region-1 (UCR1) of PDE4A long-forms is associated with markedly increased PDE4 activity [63]. Accompanying these changes in target enzyme expression and activity was a potent immunomodulatory effect of Rolipram, with changes in the SCI-induced production of a number of cytokines and chemokines. Most notable among these were an up-regulation of GM-CSF, IL-4, IL-6, IL-10 and TIMP1 and a down-regulation of MCP-1, MIP-3α, IL-1β and TNF-α. With an altered cytokine profile following SCI and Rolipram administration, a dramatic attenuation in the infiltration/activation of ED1^+^ macrophages and microglia within the lesion site was observed.

Rolipram has been used previously to elevate levels of cyclic AMP and confer neuroprotection and/or neuroplasticity after SCI, either alone [58], [62], [64] or in combination with other experimental approaches, particularly, additional pharmacological agents [62], [65], [66], cell transplantation [19], [54], [67] and/or rehabilitation [68]. When employed acutely as a neuroprotective agent after SCI, Rolipram has been demonstrated to primarily preserve white matter, with reductions in oligodendrocyte cell death and central myelinated axon loss [19], [58], [64]–[66]. These prior studies have used a variety of doses (from 0.1 to 3.0 mg/kg), routes (intraperitoneal, subcutaneous) and administration protocols (one-time injection or continuous delivery, starting as early as 30 min post-SCI and persisting for up to 2 weeks after injury) for Rolipram to mediate its neuro-protective and –plastic effects (Table 3). To date there have been no studies that have evaluated comparatively these dosing parameters for optimization of Rolipram's beneficial actions after SCI *in vivo*.

**Table 3:** Beneficial effects of Rolipram after CNS injury.

*Reference*	*Animal and CNS Injury Paradigm*	*Rolipram Administration Protocol*	*Reported Beneficial effects on Neuroprotection and/or Behavioral Outcomes*
Nikulina et al., 2004 [54]	*Animal:*Long Evan Hooded rats, 30 d old, 180–200 g*Injury Model:*C3–C4, spinal cord hemisection created using iridectomy scissors	*In vivo*- Rolipram/vehicle or vehicle only (saline and 16% DMSO) by mini osmotic pumps s.c. combined with embryonic spinal tissue implants:- 0.40 µmol/kg/h, 10 µl/h for 10 d- 0.80 µmol/kg/h, 10 µl/h for 10 d- SAC at 6–8 wk PI	*Neuroprotective:*Although neuroprotection was not assessed, Rolipram (lower dose) reduced levels of host GFAP (reduced astrogliosis) and increased numbers of serotonergic fibers in the transplant compared to vehicle only controls*Behavioral:*- Improved forelimb paw placement in Rolipram-treated, implanted animals compared to vehicle controls
Pearse et al., 2004 [19]	*Animal:*Female Fischer rats, 160–180 g*Injury Model:*Thoracic (T8) moderate contusion SCI, MASCIS Impactor	*In vivo*- Rolipram/vehicle or vehicle only (DMSO) by mini osmotic pumps s.c., with or without Schwann cell implants:- 0.5 µl/h, 0.5 mg/kg/d immediately PI, then for 2 wk PI	*Neuroprotective:*- Increased the number of spared central myelinated axons in the lateral spinal cord- Increased the number of retrogradely labeled neurons in the reticular formation and the raphe nuclei with axon projections below the level of SCI- Rolipram also prevented injury-induced reductions in cyclic AMP levels in the rostral spinal cord (for 2 wk), brainstem (for 1 wk), and sensorimotor cortex (for 1 d); reduced TNF-α at both 3 h and 6 h PI and enhanced Schwann cell myelination*Behavioral:*- Rolipram alone improved BBB subscores, foot exo-rotation, base of support and hindlimb footfall errors after SCI
Wang et al., 2006 [127]	*Animal:*Female Sprague–Dawley rats, 250–270 g*Injury Model:*Thoracic (T7) moderate contusion SCI, MASCIS Impactor	- Rolipram/vehicle or vehicle only (16% dimethylsulfoxide/PBS), mini osmotic pumps s.c.:- 1.2 mg/kg/d for 28 d	*Behavioral:*- Rolipram showed a trend for improvement on the BBB score.
Atkins et al., 2007 [20]	*Animal:*Male Sprague-Dawley rats, 270–320 g*Injury Model:*Moderate FPI (2.0±0.2atm) TBI; 3.8 mm posterior to bregma/2.5 mm lateral to the midline	- Rolipram/vehicle or vehicle only (5% DMSO/95% Saline) i.p.:- 0.3 or 3.0 mg/kg at 30 min PrI, then every 24 h PI, then SAC 30 min or 3 d later	*Neuroprotective:*- Reduced cortical contusion volume and contusion area near epicenter of injury and in penumbra region, improved total cortical neuron and CA3 neuron survival on ipsilateral side and modestly reduced β-APP deposits.- Rolipram also restored cyclic AMP levels in the hippocampus as well as total CREB levels in the parietal cortex and phosphorylated CREB levels in the hippocampus after TBI; Rolipram significantly reduced injury-induced increases in IL-1β in hippocampus and thalamus, but not parietal cortex. Significantly reduced injury-induced increases in TNF-α levels in parietal cortex and hippocampus, but not the thalamus
Kajana and Goshgarian, 2008 [128]	*Animal:*Male Sprague–Dawley rats, 3–6 mos, 295–480 g*Injury Model:*Unilateral cervical (C2) spinal cord hemisection	- Rolipram/vehicle or vehicle only (10% DMSO in saline) i.p.:- 2.0 mg/kg, 2x/d for 2 d with functional assessments at 5 d and 10 d after last dose	*Behavioral:*- Facilitated ipsilateral phrenic nerve recovery as exemplified by a recovery in motor activity (5 and 10 d) and enhanced contralateral phrenic nerve output (5 and 10 d); Rolipram-treated animals had higher respiratory rates than controls at 5 but not 10 d
Hatinen et al., 2008 [129]	*Animal:*Male Wistar rats, 3–4 mos, 262–359 g*Injury Model:*Stroke; transient occlusion of the middle cerebral artery (MCAO)	- Rolipram/vehicle or vehicle only [Macrogol 30%/0.9% NaCl (70%)] i.p.:- starting at 2 d PO for 13 d- Low-dose: a.m. 0.1 mg/kg (30 min before behavior), then p.m. 0.2 mg/kg- High-dose: a.m. 1.0 mg/kg (30 min before behavior), then p.m. 2.0 mg/kg	*Behavioral:*- Hindlimb function in the high-dose Rolipram group was associated with fewer slips on the beam walking task and high-dose Rolipram-MCAO rats resembled sham rats at 21 d PI indicating a possible delayed benefit of Rolipram- Rolipram-treated rats, however, showed decreased locomotor activity and rearing, atypical head twitches and possible hyperalgesia immediately after treatment (considered as acute side effects)
Whitaker et al., 2008 [64]	*Animal:*Female Sprague–Dawley rats, 228–267 g*Injury Model:*Cervical (C5–6) contusion SCI (180±7 kDyn), IH Impactor	- Rolipram/vehicle or vehicle only (DMSO), mini osmotic pumps s.c.:- 0.5 mg/kg/d, 0.5 ul/h for 12 h, 24 h, or 72 h PI	*Neuroprotective:*- Significantly reduced oligodendrocyte death at 24 h continuing through 72 h PI (assessed by APC immunohistochemistry)
Beaumont et al., 2009 [58]	*Animal:*Female Sprague–Dawley rats, 228–267 g*Injury Model:*Cervical (C5–6) contusion SCI (175±5 kDyn), IH Impactor	- Rolipram/vehicle or vehicle only (DMSO), mini osmotic pumps s.c.:- 0.5 mg/kg/d, 0.5 µl/h each pump for 2 wk- SAC at 5 wk PI	*Neuroprotective:*- Increased numbers of oligodendrocytes in the VLF compared to controls, though spared white matter area was unchanged- Axon conductivity through the VLF was better after Rolipram treatment than controls *Behavioral:*- Rolipram treated animals exhibited less hindlimb footfall errors than controls with no change in total number of steps, percentage of forelimb steps without footfall errors and the BBB score
Kajana and Goshgarian, 2009 [62]	*Animal:*Male Sprague–Dawley rats, 3–6 mos, 295–480 g*Injury Model:*Unilateral cervical (C2) spinal cord hemisection	- Rolipram/vehicle or vehicle only (10% DMSO in saline) i.v.:- 2 mg/kg at 1 wk PI/20 min before assessment- SAC 2 h after Rolipram	*Behavioral*- Increased both contralateral and ipsilateral phrenic nerve activity PI; electrophysiological measures indicated higher burst areas and amplitudes- Rolipram also increased cyclic AMP levels
Bretzner et al., 2010 [67]	*Animal:*Male Sprague-Dawley rats, 300–400 g*Injury Model:*Cervical (C4–5) dorsolateral funiculus crush (20 s) with fine surgical forceps, depth 2 mm	- Rolipram/vehicle or vehicle only (20 mM PBS/16% DMSO), osmotic pump s.c.:- 0.4 µmol/kg/h for 2 wk.- SAC at 5 wk PI	*Neuroprotective:*- Although neuroprotection was not assessed, Rolipram enhanced GFP-OEC cell density and axon density in the lesion site*Behavioral:*- Rolipram in combination with OEC transplants enhanced forelimb function on the cylinder test compared to vehicle only controls
Ianotti et al., 2011 [66]	*Animal:*Female Sprague Dawley rats, 220–250 g*Injury Model:*Thoracic (T8–9) moderate contusion SCI, MASCIS Impactor	- Rolipram/vehicle or vehicle only (DMSO), mini osmotic pump, s.c.:- 0.5 mg/kg/d, 0.5 µL/h, immediately PI for 2 wk	*Neuroprotective:*- Decreased lesion volume and increased spared white matter; also increased corticospinal and supraspinal axon sparing/sprouting at 5 wk PI*Behavioral:*- Rolipram improved BBB scores and subscores from 1 to 4 wk PI
Downing et al., 2012 [130]	*Animal:*Adult female athymic rats, 170–243 g*Injury Model:*Cervical (C4–6) hemisection injury using a fine scalpel blade	- Rolipram administered through a drug-eluting microfibrous patch at 2 doses:- Low dose (25 µg/ml)- High dose (500 µg/ml)	*Neuroprotective:*- Although neuroprotection was not assessed, Rolipram (low dose) increased axon presence in the lesion site, increased numbers of oligodendrocytes and reduced the degree of astrocyte reactivity*Behavioral:*- Improved Martinez forelimb open-field scores with Rolipram (low dose) from weeks 1 through 4, 6 and 8, when compared to all other animals

This table summarizes those studies that have shown beneficial effects of Rolipram on anatomical (primarily cell or tissue protection) or functional outcomes following spinal cord or brain injury. Table abbreviations: FPI: parasagittal fluid-percussion brain injury, i.p.: intraperitoneal, i.v.: intravenous, PI: post injury, PO: post operation, PrI: prior to injury, PrS: prior to sacrifice, SAC: sacrificed, TBI: traumatic brain injury, VLF, ventrolateral funiculus.

The initial evaluation of dose demonstrated that Rolipram provided neuroprotection at doses between 0.1 and 1.0 mg/kg, well within tolerated levels [53] and within the spread of doses used to demonstrate efficacy in experimental SCI models. The highest dose of Rolipram employed, 5.0 mg/kg, however, was without protective action. These results are similar to previous dose-finding studies by Yang and colleagues, in which Rolipram exhibited a bell-shaped dose effect in protecting neurons after 1-methyl-4-phenyl-1,2,3,6-tetrahydropyridine (MPTP)-induced dopamine depletion, an experimental model of Parkinson's Disease [69]. Bell-shaped responses may indicate either a dose-dependent reversal of the drug's effect on a given biological reaction or that the drug has more than one target, a high affinity target mediating a positive effect and a low affinity target associated with a negative effect, which is reached upon higher doses [70]. There is evidence for the existence of two distinct, non-inter-convertible, conformational states of PDE4 in different cell-types, with which Rolipram (and other PDE4 inhibitors) exhibit different affinities for [71], [72]. These states have been termed the high affinity binding site (HARBS) and low affinity binding site (LARBS). While the LARBS state predominates in peripheral tissues and has been associated with immune cell function and inflammation [22], [72], the HARBS state is virtually absent from the periphery though highly present in the CNS, being involved in PDE4 inhibitor activities including emesis [73], gastric acid secretion [71] as well as tracheal relaxation and bronchodilation [74]. The K*_d_* values for Rolipram binding to these two affinity states are approximately 200 and 2 nM, respectively [22]. In addition to different conformational states, PDE4 has four different gene products, A, B, C and D as well as numerous spliced variants derived from each gene product [75]. Differences in the binding affinity of Rolipram to these PDE4 enzymes [76] may also lead to concentration-specific effects. New generation PDE4 inhibitors that exhibit different binding affinities to the LARBS and HARBS compared to Rolipram [77], or have an order of magnitude greater affinity to a specific PDE4 gene product, such as PDE4D [78], may exhibit improved safety and neuroprotective efficacy.

The protective effect of Rolipram was in part due to the vehicle in which the drug was administered. Being a highly hydrophobic molecule, Rolipram exhibits poor solubility in water, requiring an organic solvent, such as Dimethyl sulfoxide (DMSO) or ethanol, for delivery in solution. Unlike previous work by our group [19] and others [58], [62], [64] in which the vehicle used for Rolipram administration, DMSO, did not have neuroprotective or functional effects, the present use of an intravenous 10% ethanol vehicle alone provided significant cytoprotection after SCI. Acute ethanol doses of less than 1.0 g/kg have been shown previously to be neuroprotective both *in vitro* [79], [80] and *in vivo* in models of ischemic stroke [81], [82] and traumatic brain injury [83], while concentrations >1.0 g/kg may produce venular cerebrovasospasm and micro-hemorrhages [84]. Ethanol's protective effects have been suggested to occur through a variety of mechanisms including: the inhibition of excitatory glutamate receptors and the facilitation of inhibitory γ-aminobutyric acid (GABA) receptors [85], increased heat shock protein expression and phosphorylation [86], decreased oxidative DNA damage, amelioration of glial cell reactivity and reduced neutrophil infiltration [81]. Caffeinol, currently in Phase I/II clinical trials for stroke [87], is a combination treatment using caffeine, a xanthine which acts as a broad spectrum PDE inhibitor, and ethanol. Caffeinol has been shown to have a potent neuroprotective action through synergism of these agents following experimental cerebral ischemia [88], [89]. Further investigation of the cellular mechanisms responsible for the synergism of PDE4 inhibitors and ethanol may provide a novel direction for therapeutic agent development of neuroprotectants after CNS injury.

When the most effective Rolipram dose was employed via different administration routes it was found that all routes offered some degree of cyto- and axo-protection. This implied that Rolipram would have a long therapeutic window after SCI, based upon the slowest pharmacokinetics associated with the different routes of administration employed. Indeed, when the therapeutic window for Rolipram was later examined Rolipram's protective effects were observed even when its initial delivery was delayed until 48 hours post-SCI. Previous studies demonstrating Rolipram's ability to provide white matter preservation after SCI employed subcutaneous minipumps that were implanted soon, within 15 minutes, after injury [19], [58], [64] for continuous delivery. The use of Rolipram in other animal models of CNS injury have shown that the therapeutic window for Rolipram's neuroprotective effects can be long, with protection observed when given even 6 hours following cerebral ischemia [90], [91] or 24 hours after quinolinic acid induced neuronal excitotoxicity [92], [93]. The ability of Rolipram to confer white, but not gray matter preservation at longer times post-SCI likely reflects the disparate phases of neuron and oligodendrocyte cell apoptosis and demyelination following contusive SCI [94]. While both apoptotic neurons and oligodendrocytes have been observed within 8 hours of SCI, from 24 hours apoptotic profiles appear largely to be those of oligodendrocytes that spread rostrally and caudally from the lesion [94], [95]. This oligodendrocyte cell death persists for at least 3 weeks post-SCI [94] and is associated with ensuing demyelination [96].

In experimental SCI models, a large number of compounds targeting a diversity of pathophysiological mechanisms have been shown to possess neuroprotective action [97]. Despite these successes, very few of these drugs have progressed to clinical evaluation; these include Methylprednisolone, Monosialoganglioside GM1 and Gacyclidine as well as more recently Riluzole, Erythropoietin and Minocycline. Like these agents, Rolipram exhibits an ability to protect both white and gray matter after SCI and has been demonstrated to provide functional benefit in a range of SCI models (Table 3). The degree of tissue preservation and functional improvement (∼2 points on the BBB score) in the current study is comparative to, or better, than that observed with the aforementioned agents in a moderate contusion injury paradigm. Using this SCI model, Mu and colleagues [98] showed that while Riluzole or Methylprednisolone did not significantly enhance tissue preservation or BBB scores, their combination did. Riluzole, however, has shown the ability to provide significant tissue preservation and pronounced improvements when used in a clip compression SCI model [99]. Minocycline, when used in a moderate thoracic contusion SCI paradigm, has been shown to improve BBB scores by ∼4 points in one study [100] and to be without effect in another [101]. Results from a recent phase II placebo-controlled randomized trial of minocycline in acute SCI reported that while efficacy was not established, there were encouraging signs that minocycline may be beneficial in specific SCI patients and should move forward to a larger Phase III trial [102]. Similar to Riluzole, Erythropoietin has shown strong neuroprotection following clip compression SCI with significant improvements in tissue sparing and functional outcome [103], [104]; results in a moderate contusion SCI model, as used in the current study with Rolipram, have been less promising [105]–[107]. Although comparative evaluation of identified neuroprotectants in standardized SCI models under similar experimental conditions may shed light on the relative effectiveness of these therapeutic approaches, differences in target injury mechanisms and the timing of these pathological events, drug administration protocols and pharmacokinetics as well as a lack of data regarding the optimization of these neuroprotectants (most effective dose or route or therapeutic window) may limit the interpretation of such work.

To better understand the mechanism(s) of Rolipram-mediated neuroprotection, it is important to examine its therapeutic target, the PDE4 family of enzymes. Previous work by Whitaker and colleagues [64] has reported that following cervical SCI, PDE4 enzymes are expressed in microglia (PDE4B) and oligodendrocytes (PDE4A, B and D). However, it was not shown whether PDE4 production or activity was up-regulated or induced after injury or whether PDE4 was affected by Rolipram treatment. In the current study, initial screening for PDE4 enzymes that exhibited increased production following SCI and were altered either at the protein or phosphorylation level by Rolipram, revealed significant increases in PDE4B1 and PDE4A5, including phospho-PDE4A5, which was abated by Rolipram. In recent work from our laboratory we have also identified SCI- and TBI-induced changes in the PDE4B2 spliced variant, which is highly expressed within activated microglia after injury [108]. Immunohistochemistry using co-staining with cell markers and a pan-PDE4A antibody showed that PDE4A was expressed in neurons and oligodendrocytes but not microglia within the normal spinal cord. Following SCI, levels of PDE4A did not change in neurons or oligodendrocytes, though an induction was observed in microglia at 24 hours. Although PDE4A was not previously observed in microglia at 72 hours following cervical SCI [64], it could be that PDE4A production is transient, being expressed only during the initial activation of microglia. Indeed many PDE4A spliced variants, PDE4A4, PDE4A7 and PDE4A10, have been detected in cells of the monocytic lineage under various conditions [109]–[111]. Unlike PDE4A, phosphorylated PDE4A was barely detectable in these cell types within the normal spinal cord. At 24 hours post-SCI, pronounced PDE4A phosphorylation was observed in neurons, oligodendrocytes and microglia, which according to immunoblotting corresponded to PDE4A5. Serine phosphorylation of PDE4A has been shown to increase the enzyme's sensitivity to Mg^2+^ and its activity [63]. In addition, the binding affinity of Rolipram for PDE4 is also dramatically increased following phosphorylation. Administration of Rolipram showed a pronounced amelioration of SCI-induced PDE4A5 phosphorylation, which would be expected to reduce PDE4 activity and elevate cyclic AMP levels [19]. *In vitro* over-expression studies have shown that PDE4A5 associates with the SRC family tyrosyl kinase LYN, is localized to the cell margin and perinuclear sites of cells [112] and can be cleaved by caspase-3 [113]. PDE4A5 expression has been identified in primary cortical neurons [114], and PDE4A5 has been shown to bind with disrupted in schizophrenia 1 (DISC1), a risk factor for schizophrenia, bipolar disorder, and major depression [115]. PDE4A5 is believed to play a role in cell survival and differentiation processes based upon cell culture studies [113], [116] though it has not been shown whether similar functions of PDE4A5 exist in neurons *in vivo*. These are the first studies to demonstrate changes in PDE4A, B and D expression and phosphorylation after traumatic CNS injury compared to the uninjured state, and their subsequent reversal following treatment with Rolipram.

PDE4 inhibition by Rolipram or other agents has been previously shown to be potently anti-inflammatory [117], [118] and this may be the main mechanism by which Rolipram provides neuroprotection following CNS trauma. We have reported that the pro-inflammatory cytokine TNF-α is reduced by Rolipram after SCI [19] and now the current work extends this inhibition to other pro-inflammatory cytokines and chemokines including MCP-1, MIP-3α and IL-1β. Conversely, Rolipram was shown to selectively up-regulate the Th2 cytokines IL-4 and IL-10, as well as increase levels of GM-CSF and TIMP1. The reduction in MCP-1 and MIP-3α, chemokines involved in the attraction of lymphocytes, monocytes and neutrophils to the site of SCI [119], [120], is likely responsible for the dramatic perturbation in ED1^+^ microglia-macrophage infiltration observed within the injured spinal cord of Rolipram treated animals compared to controls. Agents that reduce the tissue infiltration or activation of monocytes acutely after SCI have been demonstrated to afford neuroprotection and improve function [121]. The expression of Th2 cytokines has been shown to be important for largely limiting tissue damage, cavitation and dysfunction after SCI. Therapeutic delivery of IL-10 systemically has been reported to reduce tissue damage and improve functional recovery after contusive SCI [122], while inhibition of IL-4 after SCI with neutralizing antibodies markedly increased macrophage infiltration through enhanced MCP-1 expression and exacerbated tissue cavitation [123]. Rolipram also increased TIMP1, an endogenous inhibitor of metalloproteinases (MMPs), the up-regulation of which would be expected to reduce tissue damage and cavitation based upon the demonstrated detrimental effects of MMPs on blood-spinal cord-barrier (BSCB) disruption, inflammation and tissue damage after SCI [124], [125]. Collectively, these changes in cytokines after SCI and Rolipram administration demonstrate a role for PDE4 in potentiating BSCB disruption, immune cell activation and infiltration as well as the production of cytotoxic and tissue degrading molecules that would exacerbate tissue damage and neurological dysfunction after SCI.

In conclusion, the current study has optimized Rolipram as a neuroprotectant for daily, acute delivery in a clinically-relevant contusive SCI model, establishing a regimen for translation to larger animals and future human SCI clinical trials that is beneficial for both white and gray matter preservation and improved functional outcome. Although we and others have demonstrated that Rolipram is neuroprotective in experimental models of CNS injury, clinical translation of these effects, however, is not guaranteed. Rolipram's beneficial action in another neuro-inflammatory model, experimental autoimmune encephalomyelitis (EAE), was not duplicated in clinical trials for multiple sclerosis (MS) patients [126]. It remains unclear as to whether the absence of benefit was due to the deficiencies of the EAE model to replicate the clinical condition of MS or if significant species-related differences in inflammatory responses to Rolipram exist. The current studies have also for the first time characterized SCI-induced changes in the expression and phosphorylation of PDE4A, B and D spliced variants and identified how these changes after injury are affected by Rolipram. The current work highlights the importance of the vehicle used for Rolipram, ethanol, in enhancing its therapeutic action and demonstrates that Rolipram has potent immunomodulatory effects, including a switch in the Th1/Th2 balance after SCI from a Th1 to a Th2 response and the attenuation of immune cell infiltration and activation within the injured spinal cord that may be responsible for its neuroprotective effects.
